# Pyrene-Containing Polyamines as Fluorescent Receptors for Recognition of PFOA in Aqueous Media

**DOI:** 10.3390/molecules28114552

**Published:** 2023-06-05

**Authors:** Yschtar Tecla Simonini Steiner, Giammarco Maria Romano, Lara Massai, Martina Lippi, Paola Paoli, Patrizia Rossi, Matteo Savastano, Andrea Bencini

**Affiliations:** 1 Department of Chemistry “Ugo Schiff”, Università degli Studi di Firenze, Via della Lastruccia 3, Sesto Fiorentino, 50019 Firenze, Italy; yschtartecla.simoninisteiner@unifi.it (Y.T.S.S.); lara.massai@unifi.it (L.M.); matteo.savastano@unifi.it (M.S.); 2 Department of Industrial Engineering, Università di Firenze, Via Santa Marta 3, 50139 Firenze, Italy; martina.lippi@unifi.it (M.L.); paola.paoli@unifi.it (P.P.); p.rossi@unifi.it (P.R.)

**Keywords:** perfluorooctanoic acid, polyamines, fluorescent receptors, anion binding, supramolecular chemistry, zinc complexes

## Abstract

The globally widespread perfluorooctanoic acid (PFOA) is a concerning environmental contaminant, with a possible toxic long-term effects on the environment and human health The development of sensible, rapid, and low-cost detection systems is a current change in modern environmental chemistry. In this context, two triamine-based chemosensors, **L1** and **L2**, containing a fluorescent pyrene unit, and their Zn(II) complexes are proposed as fluorescent probes for the detection of PFOA in aqueous media. Binding studies carried out by means of fluorescence and NMR titrations highlight that protonated forms of the receptors can interact with the carboxylate group of PFOA, thanks to salt bridge formation with the ammonium groups of the aliphatic chain. This interaction induces a decrease in the fluorescence emission of pyrene at neutral and slightly acidic pH values. Similarly, emission quenching has also been observed upon coordination of PFOA by the Zn(II) complexes of the receptors. These results evidence that simple polyamine-based molecular receptors can be employed for the optical recognition of harmful pollutant molecules, such as PFOA, in aqueous media.

## 1. Introduction

Perfluorooctanoic acid (PFOA) and perfluorooctane sulfonic acid (PFOS) are two chemicals composed of a fully fluorinated carbon chain and a hydrophilic polar end group [1]. They are water-soluble acids that exist in the anionic form along a wide range of pH values [2,3], including neutral pH, and are widely employed in several industries, including the production of waterproof fabrics, paints and varnishes, fire-fighting foams, and cleaning compositions [4,5]. Because of the high stability of the C-F bonds, PFOA and PFOS feature a remarkable thermodynamic stability [6] making them persistent organic pollutants [7]. The wide range of their applications, their ubiquitous use, and consequent release in the environment, together with their water solubility has led to their global presence and accumulation in surface and groundwaters and soils [8]. Recent studies outline the bioaccumulative potential of PFOA and PFOS, with possible toxic effects in animals, including humans [9,10,11]. In consequence, the number of possible emitting sites is enormous (more than 100.000 in Europe [12]). Water pollution has been identified in several countries across Europe, including Austria, Denmark, France, Germany, the Netherlands, Sweden, and Italy while in the US 2858 locations in 50 states and two territories are known to be contaminated [13,14].

The strongest evidence of PFOA and PFOS toxicity is related to endocrine disorders, in particular dyslipidaemia. However, animal and epidemiological studies regarding communities exposed to contaminated drinking water and the general population established a positive association between PFOA levels and kidney and testicular cancer along with a statistically significant association between PFAS exposure and suppressed immune response [15,16].

Even though health advisory levels in drinking water have been recently established by U.S. Environmental Protection Agency (70 ng L^−1^) [17], sometimes the levels of these pollutants are higher than the reported limit. Hence, rapid and sensitive techniques for the detection of PFOA and PFOS in solution are needed [10]. Traditional non-labeled techniques have been employed to detect perfluoroalkyl substances (PFAS) in solution, which include liquid chromatography-mass spectrometry (LC-MS or HPLC-MS) [10,18,19] and gas chromatography-mass spectrometry [20]. However, they usually require expensive equipment and sample pretreatment, such as extraction, preconcentration, and derivatization steps [21,22]. These drawbacks can be overcome by methodologies based on optical measurements [23,24,25]. In the last few years, different optical approaches have been used to detect perfluorinated compounds in solution, in particular nanostructured systems, including modified Au nanoparticles [26,27] and quantum dots assays [28,29], functionalized molecular imprinted polymers [30], a DNA aptamer-based sensor [31], luminescent metal-organic framework sensors [32] or, more recently, a fluorescent imprint-and-report sensor array [33]. Less attention has been devoted to fluorescent molecular receptors for PFOA and/or PFOS detection, although fluorescent molecular chemosensors have been successfully used for a variety of anionic chemical species [34,35,36,37]. However, binding and sensing of organic anions in an aqueous solution by using molecular receptors is a hard task because of the weakness of non-covalent interactions, in particular in an aqueous solution, in which water solvation of the anionic groups can compete with their coordination. Therefore, anion receptors need to contain different binding sites appropriately disposed to establish multiple interactions with the guest species, in order to form stable complexes even in aqueous media. [38]. Accordingly, only a few examples of molecular sensors have been reported for PFOA or PFOS, such as a fluorescent perylene diimide derivative [39], a calix[4]arene functionalized at the lower rim with amide groups, and fluorous ponytails [40] and an erythrosine B-based sensor [41]. Polyamines are among the most interesting scaffolds to develop supramolecular chemosensors for the detection of anionic species in aqueous media. Protonation of polyamines, which can occur even at a neutral pH value in water solution, leads to the formation of polyammonium cations, able to bind anionic substrates thanks to the formation of energetically stabilizing salt bridging (simultaneous H-bonding and electrostatic) interactions with the negatively charged functionalities of the guest [34,35,36,37,42,43,44,45,46,47,48,49,50,51,52,53,54]. Enhanced selectivity and optical sensing can be obtained with the introduction of a fluorogenic unit that responds to the presence of the analyte with a change in its photophysical properties. An ‘added value’ of polyamine receptors in anion binding and sensing is their ability to form stable metal complexes in aqueous solution, in particular with transition metals, some of which are emissive. This is the case of Zn(II) complexes, which have also been used as optical chemosensors for anions, exploiting the ability of Zn(II) to expand its coordination environment to bind exogenous substrates., which may result in a change of the emission properties of the complex [45,55,56,57,58]. Nevertheless, no attempt about their use for PFOA or PFOS binding and/or sensing has yet been reported.

In this panorama, we thought that coupling in close proximity within a receptor structure a hydrophilic polyamine unit with a large fluorescent aromatic moiety could constitute a simple approach to developing new efficient chemosensors for PFOS and/or PFOA. While the polyamine unit can ensure solubility in aqueous media and, in its protonated form, salt bridging contacts with the carboxylate or sulphonate groups of the substrates, a fluorescent aromatic moiety can give hydrophobic interaction with the perfluoroalkyl chains and, at the same time, can be used as signaling reporter for PFOA and/or PFOS. To this purpose, we joined via a methylene bridge a diethylen- or a dipropylen-triamine unit, among the most used binding units in both cations and anion coordination [46,54,59,60], with a pyrene unit, a well-known large and hydrophobic aromatic moiety, often used in fluorescent chemosensors for anions.

**Scheme 1:**
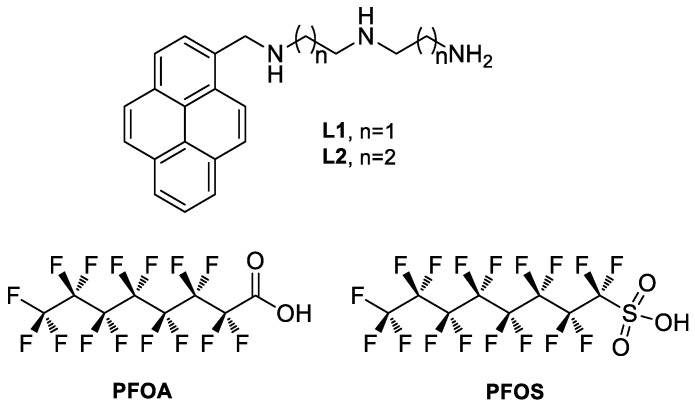
Fluorescent polyamines receptors (**L1** and **L2**) and target analytes.

The purpose of the present paper is the analysis of the resulting receptors (**L1** and **L2** in Scheme 1), which can be obtained via a simple synthetic procedure, and their Zn(II) complexes as fluorescence probes for PFOA and PFOS in aqueous media [39].

## 2. Results and Discussion

### 2.1. Synthesis of ***L1*** and ***L2***

**Scheme 2:**
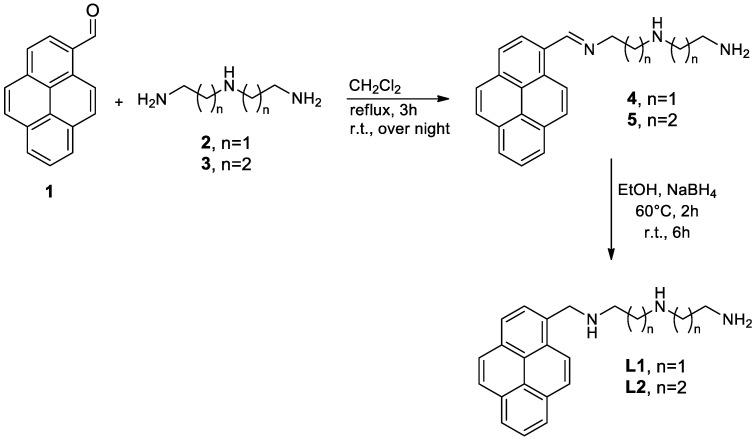
Synthetic pathway to obtain **L1** and **L2** receptors.

The molecular receptors **L1** and **L2** were synthesized by a two-step reductive amination reaction (Scheme 2), modifying a previously reported procedure for similar compounds [61]. The reaction of the carbonyl group of pyrene-1-carbaldehyde (**1**, for compound numbering, see Scheme 2) with primary amine of bis(3-aminopropyl)amine (**2**) or bis(3-aminopropyl)amine (**3**) in dry CH_2_Cl_2_ leads to the formation of the imine form of the receptors (**4** and **5**). In this step, an excess of triamine is needed to avoid the formation of the symmetric product, in which each terminal amine group is linked to a pyrene fragment. The reaction of the imine **4** or **5** with an excess of NaBH_4_ leads to the reduction of the imine bond and the formation of the receptors **L1** and **L2** as not-protonated amines. To obtain an easy-to-handle product, the receptors were precipitated as hydrochloride salts by treatment of an EtOH solution with 37% HCl of **L1** or **L2**.

### 2.2. Acid-Base Properties

**Figure 1:**
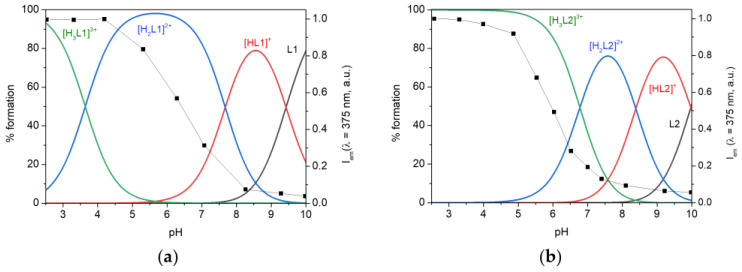
Fluorescence emission at 375 nm (black squares) of (**a**) **L1** and (**b**) **L2** superimposed to the distribution diagrams of the protonated species present in solution ([**L1**] = [**L2**] = 5 × 10^−4^ M), calculated on the basis of the equilibrium constants in Table 1 (H_2_O/EtOH 50:50 (*v*/*v*), 0.1 M NMe_4_Cl, 298.1 ± 0.1 K).

The determination of the protonation behavior of polyamine receptors is a necessary preliminary study for the analysis of their binding ability toward anionic substrates. To this purpose, potentiometric titrations have been used to determine the protonation constants of the two receptors, identifying the species formed in solution at different pH values. Because of the low solubility of both receptors in pure water at the concentration used in the potentiometric measurements, the titrations have been performed in an H_2_O/EtOH (50:50 *v*/*v*) mixture. Protonation constants values, displayed in Table 1, are in the range between 9.44 and 3.64 for **L1** and between 9.97 and 6.78 log units for **L2**. Both **L1** and **L2** feature a quite high constant relative to the first protonation equilibrium, likely attributable to the protonation of the terminal primary amine group, more basic than secondary ones in aqueous media [62]. In the case of **L1**, a marked drop in the protonation constants is observed from the second to the third pronation equilibrium. While in the H_2_**L1**^2+^ species, the two acidic protons can be localized on the primary amine group and on the benzylic one, adjacent to the pyrene unit and therefore they would be separated by a not protonated amine group, thus minimizing the electrostatic repulsions between ammonium groups, in H_3_**L1**^3+^ the three ammonium groups are necessarily contiguous. The consequent higher electrostatic repulsion leads to a markedly lower value of the third protonation constant of **L1**. **L2** is slightly more basic than **L1** in the second, and, overall in the third protonation step, (log *K*_1_ = 3.64 for **L1** and log *K*_1_ = 6.78 for **L2**), probably because of the increased distance between the protonable sites in **L2**, which reduces the electrostatic repulsion between positive charges. Distribution diagrams of the protonated species present in solution, derived from the obtained protonation constants Figure 1 shows that in the case of both receptors, the most abundant species at neutral pH value is the diprotonated form H_2_**L**^2+^ (**L** = **L1** or **L2**), accompanied by minor amounts of the H**L1**^+^ and H_2_**L2**^2+^ species in the case of **L1** and **L2**, respectively

**Figure 2:**
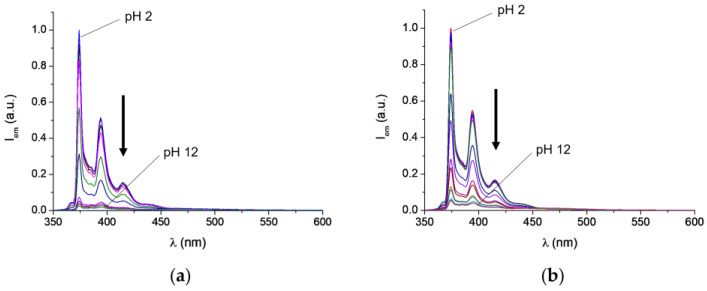
Fluorescence spectra, represented in different colors, of (**a**) **L1** and (**b**) **L2** at different pH values in H_2_O/EtOH 50:50 (*v*/*v*) ([**L1**] = [**L2**] = 10^−5^ M, λ_exc_ = 340 nm, 298 K). Arrows outline the emission decrease as the pH increases from 2 to 12.

The absorption spectra of both receptors, in H_2_O/EtOH 50:50 (*v*/*v*) mixture, show the typical pyrene structured band featuring three peaks at around 343, 328, and 314 nm. As shown in Figure S13 (Supplementary Materials, SM) the ligand absorption spectra are almost independent of the protonation equilibria involving the triamine chain, a behaviour similar to that observed for analogous triamine-based fluorescent containing anthracene [63] or phenanthroline units [64]. Conversely, the fluorescence emission spectra show a marked pH dependence (Figure 2). At strongly acidic pH values, both **L1** and **L2** show the typically structured emission band of monomeric pyrene between 370 and 450 nm (λ_exc_ = 340 nm). By increasing pH, the fluorescence emission intensity starts decreasing above pH ca 5, to achieve an almost constant value at pH > 9. At this pH value, the two receptors become basically not emissive. On the other hand, for pH greater than 9, the two receptors are in their not protonated, or, at most, mono-protonated forms, in which an acidic proton is likely localized on the terminal primary amine group. Therefore, the emission is likely inhibited by a photoinduced electron transfer (PET) effect from the aliphatic amine, in particular the benzylic amine group adjacent to the pyrene unit, to the excited fluorophore, as often observed in polyamine receptors bearing fluorescent moieties [63,64,65]. These changes can be conveniently explained in terms of different protonated species present in the solution. Superimposition of the distribution curves of the protonated species of **L1** and **L2** with the emission values of the receptor as a function of pH (Figure 1) points out that the emission intensity increases with the formation of the diprotonated species of **L1**, while in the case of **L2_,_** protonation of all three nitrogens is required to restore the original emission of pyrene. In the case of **L1**, the increased emission of the H_2_**L1**^2+^ species may be related to protonation of the benzylic amine group, adjacent to the pyrene unit, which may result in the most effective quencher of the excited fluorophore. The different behavior found for **L2** may be attributed to the higher basicity of **L2**, which makes this ligand an overall better electron donor with respect to **L1**. As a consequence, in the case of **L2**, only full protonation of the triamine chain inhibits the PET effect to excited pyrene.

### 2.3. Binding and Fluorescence Sensing of PFOA by ***L1*** and ***L2***

**Figure 3:**
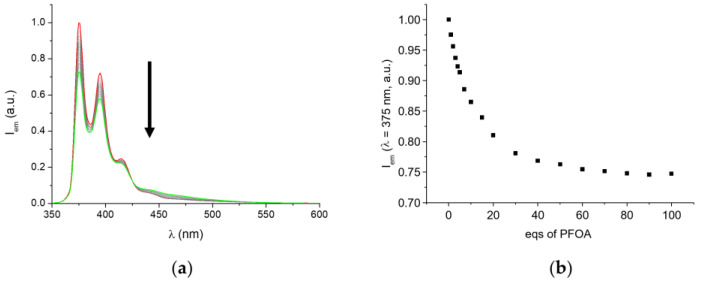
(**a**) Fluorescence emission spectra of **L1** at pH 7 (0.005 M TRIS buffer) in H_2_O/EtOH 50:50 (*v*/*v*) in the presence of increasing amounts of PFOA (spectra recorded in the absence and in the presence of 100 equivs. of PFOA are in red and green colors, respectively), [**L1**] = 10^−5^ M, λ_exc_ = 340 nm) and (**b**) plot of the fluorescence emission of **L1** at 375 nm (arrow outlines the emission decrease as the equivs. of PFOA added increase from 0 to 100).

To investigate the binding abilities and optical sensing of the two triamine receptors toward PFOA and PFOS, we performed UV-vis spectrophotometric and fluorescence emission titrations at 298 K by adding increasing amounts of the two analytes to solution of **L1** or **L2** in H_2_O/EtOH mixture (50:50 *v*/*v*) buffered at pH 7 (0.005 M TRIS buffer), normally chosen as reference pH value for a possible application of the probes in a real environmental matrix. In these conditions, both receptors show a low emission intensity (Figure 2), which is, however, further decreased by the addition of PFOA to both **L1 (**Figure 3) and **L2** (Figure S15). The addition of 10 equivs. of PFOA to a solution of **L1** and **L2** induces an almost linear slight decrease of the emission (*ca* 10% and 6% in the case of **L1** and **L2**, respectively. Further addition of PFOA gives rise to a smoother emission reduction of the two receptors, which result in *ca* 25% and 13% decreased in the presence of more than 80 equivs. of the analyte. The low emission decrease observed may be related to the presence in solution of poorly emissive species, [H**L1**]^+^ and [H_2_**L2**]^2+^ in the case of **L1** and **L2**, respectively. This applies in particular to **L2**, for which the [H_2_**L2**]^2+^ species is the most abundant species in solution at pH 7.

Nevertheless, the analysis of the spectrofluorimetric data with the HYPSPEC [66] program points out the formation of complexes with receptor to substrate 1:1 stoichiometry featuring estimated apparent binding constants, at pH 7.0 (ligand speciation not analytically considered), for the addition of PFOA to **L1** or **L2** of 5.1(1) and 4.8(1) log units, respectively. Detection limits (LOD) of 1.2 µM and 6.8 µM were estimated for PFOA sensing by **L1** and **L2**, respectively, in these experimental conditions.

Differently from PFOA, the addition of PFOS, even in large excess, does not induce any change in the fluorescence emission of **L1** or **L2** at pH 7 (TRIS buffer). This result can appear surprising, considering that the PFOS, at a neutral pH value, is in its negatively dicharged form, which would ensure a higher overall electrostatic interaction with the polyammonium chain of the two receptors. On the other hand, the sulphonate group possesses a lower H-bonding acceptor ability than the carboxylate one. The observed poor effect of PFOS with respect to PFOA on the emission of **L1** and **L2** is in accord with the relevance of directional salt bridging interactions, which involves the formation of strong H-bonding interactions occurring between the carboxylate group of PFOA and the receptor polyammonium chains with respect to an overall electrostatic interaction. The latter is likely to be prevalent in the case of PFOS and not sufficient for the formation of stable adducts with the protonated receptors.

**Figure 4:**
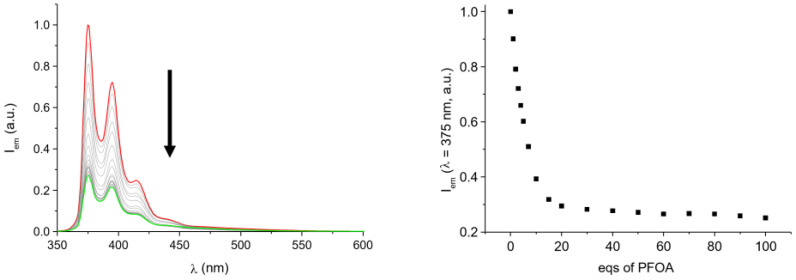
(**a**) Fluorescence emission spectra of **L1** at pH 4 in H_2_O/EtOH 50:50 (*v*/*v*) in the presence of increasing amounts of PFOA (spectra recorded in the absence and in the presence of 100 equivs. of PFOA are in red and green colors, respectively), [**L1**] = 10^−5^ M, λ_exc_ = 340 nm) and (**b**) plot of the fluorescence emission of **L1** at 375 nm (arrow outlines the emission decrease as the equivs. of PFOA added increase from 0 to 100).

To clarify the relevance of the protonation state of the polyamine chain in the formation of the complexes and the consequent changes in the emission properties of the fluorophore, we also performed UV-vis and fluorescence emission titration at pH 4. At this pH value, the two receptors are present in solution as fully emissive species [H_3_**L1**]^3+^ and [H_2_**L1**]^2+^ in the case of **L1** and [H_3_**L2**]^3+^ in the case of **L2** (see Figure 1). The absorption spectra of **L1** and **L2** are basically not affected by the presence of PFOA (Figures S17 and S18). Conversely, the emission spectra of pyrene are remarkably quenched by the addition of PFOA to the solution of **L1** and **L2** at pH 4. In the case of **L1** (Figure 4), the fluorescence emission intensity is reduced by *ca* 50% upon the addition of 10 equivs. of PFOA. Further addition of the analyte induces a minor decrease of the emission, which is reduced in the presence of 100 equivs. of PFOA at ca 30% of its original emission. A similar behavior is found in the case of **L2** (Figure S18). In this case, however, the emission decrease induced by PFOA is somewhat lower, with a 25% decrease of the original emission of the receptor in the presence of 10 equivs. of PFOA and a final 40% decrease after the addition of more than 70 equivs. of the analyte. In these conditions, treatment of emission spectra with the HYPSPEC program points out the formation of 1:1 adducts, with conditional stability constants of 5.6(1) and 5.3(1) log units and detection limits (LOD) of 0.22 and 0.66 µM for the **L1** and **L2** complexes, respectively. As already observed at pH 7 addition of PFOS to an **L1** and **L2** solution gives an almost negligible quenching of the pyrene emission (*ca* 4% in the presence of 100 equivs. of PFOS, Figure S19). The LOD values are not exceptional. However, the sensibility of the chemosensors can be, in perspective, increased by their incorporation in nanostructured materials, such as silica or organic polymeric nanoparticles. In fact, it has been demonstrated that cooperative energy transfer processes between probes, assembled at close distances one from each other, can greatly improve their detection performances [67].

Of note, the stability of the adducts formed by both receptors is higher than most of the examples of host molecules able to bind PFOA, such as *β*-cyclodextrin receptors, in which PFOA is encapsulated within the host cavity [68], a tripodal fluorous amide host [69] or calix[4]arenes functionalized at the lower rim with amide groups and fluorous ponytails [40]. Only a recently reported guanidinocalix[5]arene, able to envelop PFOA within its cavity [8], shows the higher binding ability for PFOA (the formation constants of the adduct with PFOA is 1.3 × 10^7^). The latter displays a higher sensing ability for PFOA with respect to **L1** and **L2** (LOD 26.4 nM), but does not discriminate between PFOA and PFOS. From this point of view, the ability of **L1** and **L2** to selectively bind PFOA over PFOS is a peculiar characteristic of these receptors in the panorama of molecular hosts for these pollutants. As a preliminary investigation of the effects of interfering agents on the emission of the PFOA complexes with **L1** and **L2**, we analyzed the fluorescence response of **L1** and **L2** in the presence of PFOA (100 equivs.) and an excess (200 equivs.) of a few model compounds, which, for their anionic nature, can be considered suitable candidates as interfering agents. We choose 1,3,5-nitrophenol, benzenesulphonic acid, 4-nitro-benzoic acid, and simple metansulphonic acid, which are in anionic form at neutral pH value, and may represent good candidates as organic interfering pollutants. Fluorescence measurements reported in Figure S28a,d point out that the presence of the selected interfering agents does not affect the emission of **L1** and **L2** adducts with PFOA. No changes in the emission properties and sensing abilities of **L1** and **L2** for PFOA are also found when environmentally relevant cations (Na^+^, K^+^, Ca^2+^ and Mg^2+^) or anions (Cl^−^, Br^−^, ClO_4_^−^, NO_3_^−^, HPO_4_^2−^ and SO_4_^2−^) are present in solution in excess with respect to PFOA (Figures S28b,c,e,f and S29b,c,e,f).

**Figure 5:**
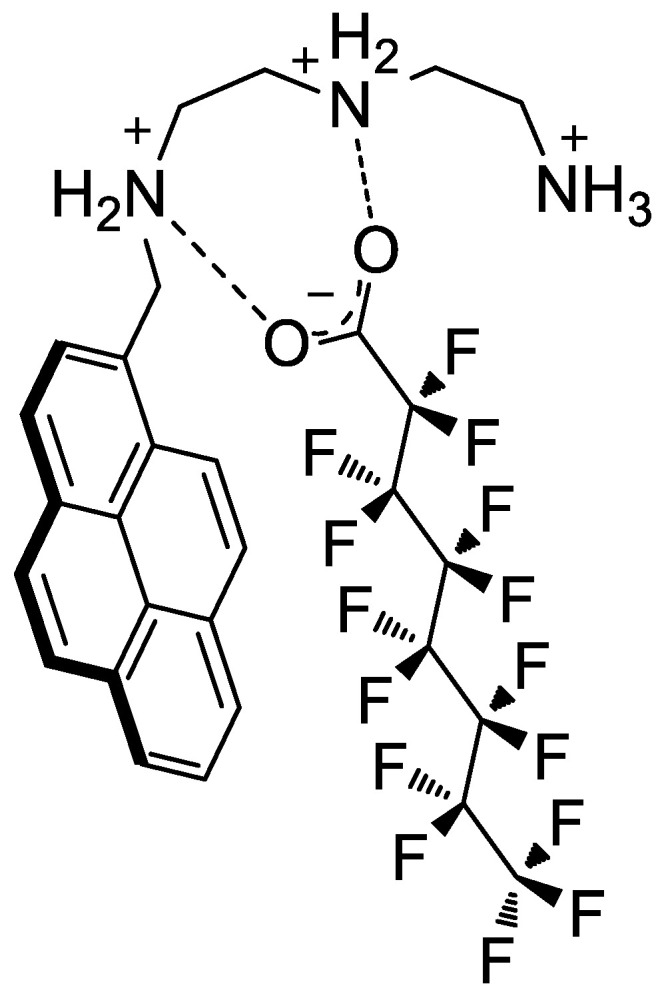
Proposed structure of the PFOA adduct with **L1**.

The higher addition constants observed at pH 4 for the addition of PFOA, in the anionic form at this pH value, to **L1** and **L2** with respect to those found at pH 7 would suggest that the host-guest interaction involves salt bridging NH_2_^+**…**−^OOC contacts between the carboxylate group of PFOA and the ammonium groups of the receptor, which, in turn, possess a higher protonation degree, i.e., a larger number of ammonium groups, at the lower pH values. This interaction mode would also explain the quenching effect observed upon PFOA binding. Interaction of protonated amine groups with anionic moiety via salt bridging implies partial sharing via H-bonding of an acidic proton, originally located on the amine group, with the anionic moiety of PFOA, thus favoring, in the excited state, electron transfer (PET) processes from the ammonium groups of **L1** or **L2** to the pyrene unit, inducing fluorescence emission quenching. To evaluate the influence of temperature in PFOA binding to the two chemosensors, the fluorescence titrations of **L1** and **L2** in the presence of increasing amounts of PFOA at pH 4 were also repeated at 288 K and 308 K (Figures S20 and S21), without evidencing appreciable differences from the binding experiment performed at 298 K. The receptors show a very similar binding ability for PFOA at three temperatures. However, at the lower temperature (288 K) a slightly higher fluorescence decrease is observed, with a consequent somewhat higher host-guest association constant. This result is in agreement with the hypothesis that the observed emission decrease is related to static quenching upon host-guest interaction via electrostatic contacts and H-bonding. A sketch of the proposed interaction mode is shown in Figure 5. Lower temperatures facilitate the formation of the complex, increasing its stability [65] and leading to a somewhat lower emission intensity, as actually observed in our measurements. Apparent constant of 5.8(1) and 5.5(1) log units for the addition of PFOA to **L1** and **L2,** respectively, at 288 K were calculated by data treatment with the Hypspec program. Slightly lower binding constants were obtained at 308 K, with values of 5.5 log units, in the case of **L1**, and 5.2 log units, in the case of **L2**.

Despite its lower charge at both pH 4 and 7, **L1** appears to be a better PFOA binder than **L2**, suggesting that the shorter ethylenic chains of **L1** can favor the formation of stronger hydrogen bonds between the COO^−^ polar hedge and two contiguous protonated amine groups of this receptor. Finally, the absence of any effect on the receptor fluorescence emission in the presence of PFOS, whose sulphonate moiety has poor H-bonding acceptor ability, further confirms the relevance of directional NH_2_^+**…**−^OOC H-bonding interactions in the formation and stabilization of the adducts.

Potentiometric measurements confirmed that no interaction occur between the two receptors with PFOS. In the case of PFOA, precipitation is observed at an acidic pH value (below pH 7) in the condition of the potentiometric titrations (receptor concentrations *ca* 1 × 10^−5^ M, 0.1 M NMe_4_Cl), likely due to the formation of an insoluble adduct between this analyte and the two receptors, preventing the determination of the stability constants of the adducts formed.

While this scenario reasonably depicts the role of the polyamine chain in PFOA binding, possible hydrophobic or C-F^…^π interactions involving the perfluoroalkyl chain of guests and the pyrene units of **L1** and **L2** could participate in complex stabilization. To this purpose, we performed ^1^H NMR titrations on **L1** and **L2** in the presence of increasing amounts of PFOA and PFOS at pH 4 and 7.

**Figure 6:**
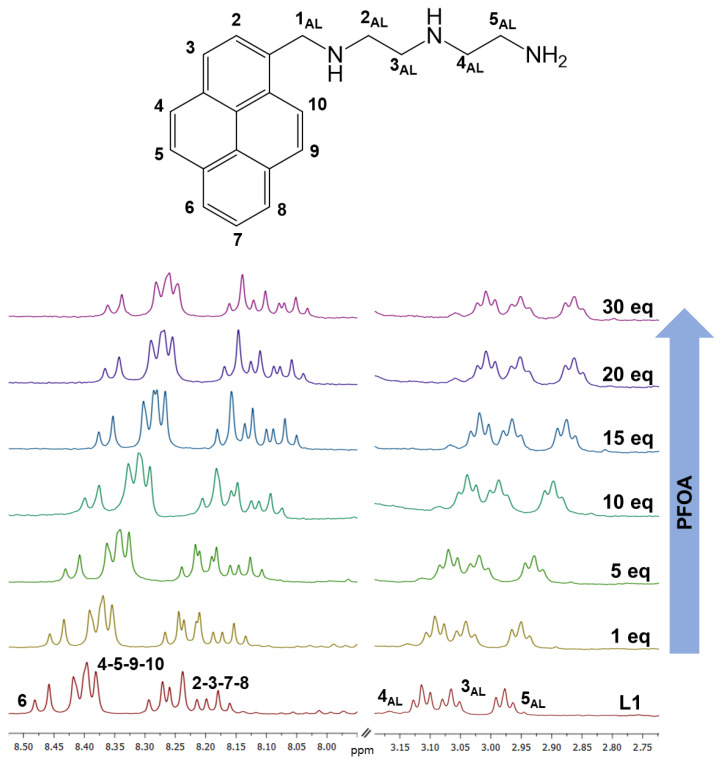
^1^H NMR spectra of **L1** at pH 4 D_2_O/CD_3_OD 40:60 (*v*/*v*) in the presence of increasing amounts of PFOA at 298 K. The signals of the 1_AL_ and 2_AL_ methylene groups cannot be observed due to overlapping with the methyl group of methanol.

The receptors and their complexes show a poor solubility in water/ethanol mixture in the condition of ^1^H NMR measurements (receptor concentration *ca* 10^−2^ M). We partially solved this drawback by performing ^1^H NMR titrations at pH 7 and 4 in D_2_O/CD_3_OD 40:60 (*v*/*v*) mixture. However, **L2** suffers low solubility even in this medium at pH 4, and therefore, the system **L2**-PFOA was analyzed only at pH 7. In the case of **L1**, the addition of increasing amounts of PFOA, up to 20 equivs., to a solution of the receptor induces a progressive upfield shift both at pH 7 and 4 (see Figure 6 and Figure S27 for the spectra at pH 4 and 7 respectively) of the ^1^H resonances of the methylene groups 3, 4 and 5, all of which show similar shifts (0.5–0.6 ppm and 0.9–0.1 pH 7 and 4, respectively). The addition of more than 20 equivs. of PFOA does not significantly affect the spectra. The signals of the 1_AL_ and 2_AL_ methylene groups (-CH_2_- groups adjacent to the benzylic amine group; for receptor numbering see Figure 6 and Figure S25) at pH 4 and 7 and of 1_AL_ at pH 4 overlap with the methyl group of methanol, precluding the determination of their chemical shifts. Considering that protonation of aliphatic amines is associated with a downfield shift of the signals of the adjacent methylene units [62], the observed upfield shift can be reasonably ascribed to an increased electronic density on the amine groups upon PFOA binding, likely due to sharing, via H-bonding, of the positive charge of ammonium groups with the carboxylate moiety of PFOA, in keeping with the suggestions derived from the fluorescence emission measurements. The higher upfield shift observed at pH 4 is in agreement with the increased number of ammonium groups available for salt-bridging interactions with the carboxylate group of PFOA. A similar upfield shift of the aliphatic signals is also found by adding increasing amounts of PFOA to the solution of **L2**, although in this case, the poor solubility of the **L2** at pH 4 prevents analysis of the spectra (Figure S26). However, at pH 7, a greater shift is observed for the 2_AL_ (for receptor numbering see Figure S26) adjacent to the benzylic amine groups, while minor shifts are observed for the remaining signals. This would suggest a stronger salt-bridging interaction with the amine group adjacent to the pyrene unit. Unfortunately, as in the case of **L1**, the signal of the 1_AL_ cannot be observed, due to overlapping with the methyl group of methanol. Besides the shift of the aliphatic protons, ^1^H NMR spectra of both **L1** and **L2** also point out an upfield shift of the aromatic resonances of pyrene, more marked for **L1** at pH 4 (see Figure 6 and Figure S26 for **L1** and **L2** at 7), in keeping with a stronger host-guest interaction at this pH. In an attempt to clarify the role of the pyrene unit in PFOA binding, we also performed ^19^F titrations by adding increasing amounts of **L1** or **L2** to 10^−2^ solutions of PFOA. Unfortunately, in these conditions, precipitation at pH 4 prevents spectral analysis, while the spectra recorded at pH 7 (see Figure S28) do not show a significant shift of the ^19^F resonances. This result could indicate the absence of strong C-F^…^π interactions. On the other hand, pyrene is a rather electron-rich system, not so prone to strongly interact with the electron-rich fluorine atoms of PFOA. In this context, the shift observed for the pyrene ^1^H NMR signal upon complex formation could be due to hydrophobic effects, i.e., changes of solvation of pyrene upon binding of PFOA.

Interestingly, ^1^H titration carried out by adding PFOS to the solution of **L1** at pH 4 in D_2_O/MeOD 40:60 (*v*/*v*) mixture, shows negligible shift for both the aliphatic methylene groups and the pyrene protons, confirming the suggestion derived for fluorescence emission analysis. Similarly, the addition of PFOS to a solution of **L1** at pH 7 and to the solution of **L2** at pH 4 and 7 did not induce any relevant shifting of the signals of the receptors.

### 2.4. Zn(II) Complexation

**Figure 7:**
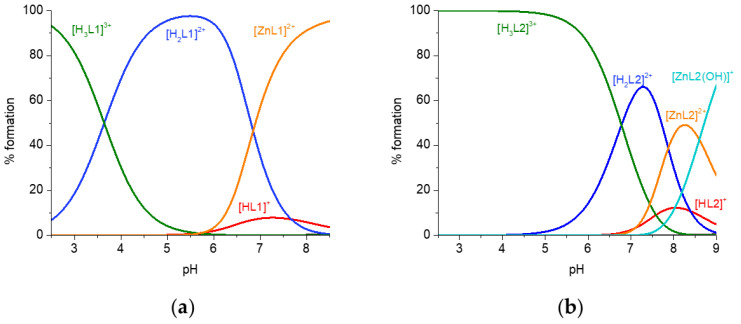
Distribution diagrams of the Zn(II) complexes formed by **L1** (**a**) and **L2** (**b**) in H_2_O/EtOH (50:50 *v*/*v*) (0.1 M NMe_4_Cl, 298.1 ± 0.1 K).

As expected, both **L1** and **L2** form stable 1:1 complexes with Zn(II) that can be isolated as solids from H_2_O/EtOH 1:1 solutions. Potentiometric titrations have been also used to determine the binding constant of the receptors toward the Zn(II) cation. The measurements have been carried out in an H_2_O/EtOH 50:50 (*v*/*v*) mixture in the presence of 0.8 equivs. of metal ions, and the explored pH range was 2.5–9 for **L2** and 2.5–8.5 for **L1** (above these pH values, precipitation of the metal complexes prevents the analysis of the systems). The binding constants of both receptors are reported in Table 2, while Figure 7 displays the distribution diagrams of the species present in the solution.

**Figure 8:**
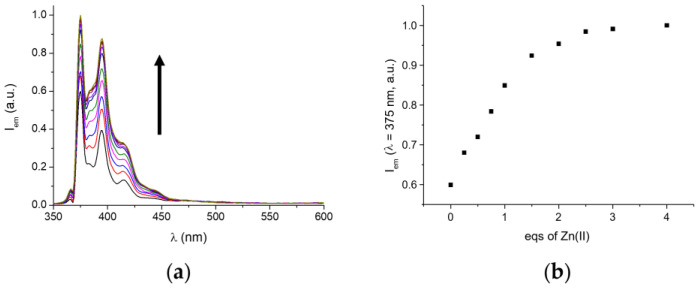
(**a**) Fluorescence emission spectra, reported in different colors, of **L1** at pH 8 (0.005 M TRIS buffer) in H_2_O/EtOH 50:50 (*v*/*v*) in the presence of increasing amounts of Zn(II) ([**L1**] = 10^−^^5^ M, λ_exc_ = 340 nm) and (**b**) plot of the fluorescence emission of **L1** at 375 nm (arrow outlines the emission increase as the equivs. of Zn(II) added increase from 0 to 4).

Both **L1** and **L2** are able to form Zn(II) complexes in neutral or slightly alkaline pH conditions. The higher formation constant of **L1** (log K = 8.69) with respect to that of **L2** (log K = 7.88) is in agreement with the lower ability of the dipropylentriamine chain in first-row transition metal coordination than diethylentriamine [70,71]. The stability of the **L1** and **L2** complexes is similar or somewhat lower than that found for corresponding complexes with diethylentriamine (den) and dipropylentriamine (dpt), respectively, although the stability of the latter complexes was determined in water. Similarly to the present case, however, the stability of the Zn(II) complex with den is ca 0.8 log units higher than that of the complex with dpt. In the case of **L2**, a mono-hydroxylated species [Zn**L2**(OH)]^+^ is also found in solution, as often observed for Zn(II) complexes in which the coordination sphere of the metal is not saturated by ligand donor atoms. Similar species most likely exists also for **L1**, yet complex precipitation prevents a confident determination of such species. We decided to compare the emission of the Zn(II) with **L1** and **L2** at pH 8. At higher pH values, in fact, precipitation could prevent a correct analysis of the emission phenomena. At pH 8, the Zn(II) complexes of **L1** and **L2** are the most abundant species present in solution, although in the case of **L2** a not trascurable amount of the receptors is also present in solution in its metal-free form. As discussed above, both receptors are poorly emissive at pH 8 (TRIS buffer) in water/ethanol solution (50:50 *v*/*v*). As often observed for Zn(II) complexes with fluorescent polyamine ligands, Zn(II) binding to both **L1** and **L2** results in the formation of emissive 1:1 complexes. When increasing amounts of Zn(II) is added to a solution of **L1** at pH 8, the fluorescence emission linearly increases up to a 1 metal-to-ligand molar ratio (R), achieving a constant value for R > 2 (see Figure 8b), confirming the formation in a solution of complexes with 1:1 metal to ligand stoichiometry. The metal cation complexation by the three nitrogen atoms of the polyamine chain prevents the photoinduced transfer of the benzylic nitrogen lone pair’s electron, which inhibits the PET effect.

A similar behavior is also observed in the case of **L2** (Figure S31), although the emission enhancement is less pronounced in this case, likely due to the presence in solution of a greater amount of receptor not bound to the metal and/or to present in **L2** of a weakly bound nitrogen atom which can thus partially quench pyrene fluorescence via the PET effect. However, the linear relationship between the measured fluorescence intensity and added equivs. of Zn(II) up to an R value of ca 1, suggests the formation of complexes with 1:1 stoichiometry.

### 2.5. Description of the Crystal Structure of [Zn***L2***(Cl)](ClO_4_)

**Figure 9:**
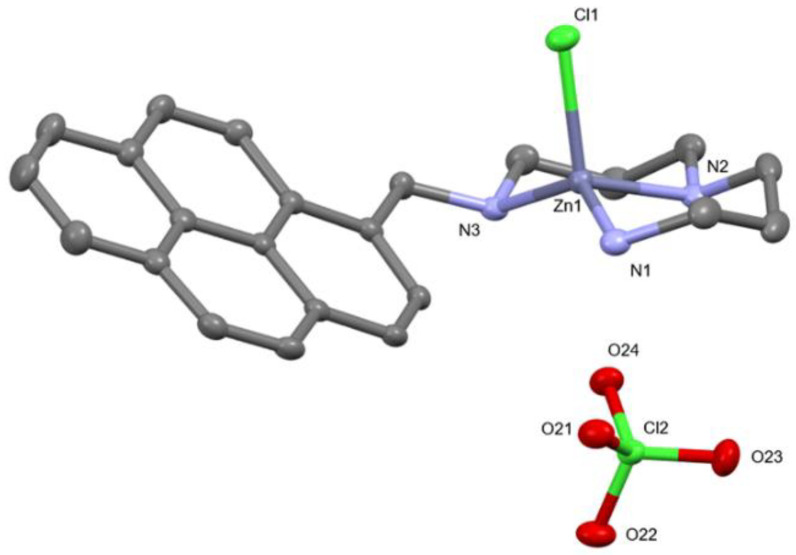
ORTEP view of the asymmetric unit of **6** with adopted labelling scheme. Displacement ellipsoids are drawn at the 50% probability level. Hydrogen atoms are not shown for the sake of clarity.

Slow evaporation of an H_2_O/EtOH (*v*/*v*) solution containing **L2** and ZnCl_2_^.^6H_2_O in 1:1 molar ratio in the presence of an excess of NaClO_4_ (10 equivs.). In the asymmetric unit of [Zn**L2**Cl](ClO_4_) (compound **6**) one [Zn**L2**(Cl)]^+^ cation and one ClO_4_^−^ anion are present. The Zn(II) metal ion is bound by the three nitrogen atoms of the polyamine, while a chloride ligand completes the tetracoordinated environment (see Figure 9). The coordination geometry appears distorted (τ4 = [360 − (α + β)]/141 = 0.827, with α and β two largest metal-centered angles in the four-coordinate species) [72] and it is overall close to a trigonal pyramidal arrangement (expected τ4 values for non-distorted limit geometries are 1 for the tetrahedron, 0.85 for trigonal pyramid, 0 for square planar). Bond distances and angles defining the metal coordination environment are reported in Table S1. Trigonal pyramidal arrangements with one large N-Zn-N angle (here 134.19(7)°, N3-Zn1-N1, Table S1), are common for N4 tripodal ligands, although they have also been observed for constrained triaminic fragments, e.g., belonging to small ([9]aneN_3_) or larger macrocyclic derivatives unable to provide further donor atoms. In the present case, steric hindrance from the large pyrene pendant is likely to subtend to the adopted coordination geometry.

The non-coordinating perchlorate counteranion is far from the metal centre (Zn1^…^O24 3.199(1) Å), yet collinear to the Cl-Zn bond (Cl1-Zn1-O24 177.43(3)°): this long electrostatic interaction virtually completes a trigonal bipyramidal environment. The presence of outer sphere donor-acceptor interactions has been observed in Zn(II) polyamine complexes and generally happens along the expected direction for the addition of a further inner sphere ligand. For instance, prototypical square planar Zn(II) porphyrin complexes, when they interact with a fifth out-of-sphere oxygen donor, do so axially (average N-Zn^…^O angle 92 ± 6, average Zn…O distance 2.5 ± 0.2 Å, sum runs on 74 hits from 61 CSD deposited crystal structures). Mononuclear examples of tetracoordinated Zn(II) centres bound to three nitrogen donors further showing out of sphere Zn^…^O contacts, as in our case, are rare, yet the FOJTAZ [73] and IJAMEN [74] examples also show the outer sphere ligand formally completing a trigonal bipyramidal environment, as in **6**. It must be also stated that the perchlorate anion is firmly hold in that position also thanks to NH^…^O salt bridges (O21^…^N1 2.956(2) Å, H1NB^…^O21 2.07(2) Å, N1-H1NB-O21 168(2)°, and the longer O24-N2 3.128(2) Å, H2N^…^O24 2.47(2) Å, N2-H2N-O24 133(2)°).

**Figure 10:**
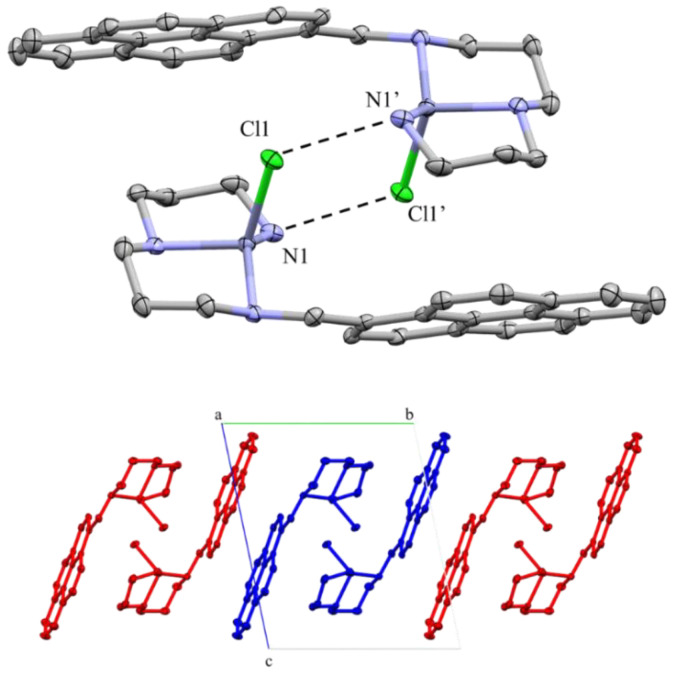
(**Top**): view of hydrogen-bonded dimers formed by the [Zn(**L2**)Cl]^+^ structural units. (**Bottom**): a central H-bonded dimer (blue) interacts with its prime neighbors via π-π stacking to form columns growing along the b axis.

The overall packing seems to be regulated according to two kinds of interactions (Figure 10). Complexes arrange themselves in dimers held together by double mutual Cl^…^HN hydrogen bonds (Cl1^…^N1′ 3.350(2) Å, H1NA^…^Cl1 2.53(3), N1-H1NA-Cl1 151(2)°), which prompts a head to tail disposition among them (Figure 10). This leaves the flat π surface of pyrene completely exposed towards the outside, resulting in an overall columnar arrangement, giving rise to typical π-stacked columns growing along the b crystallographic axis (pyrene-pyrene mean C planes distance 3.527 Å). Columns of complexes are held together by scant interactions, among which a Cl^…^HC dipolar contact (Cl1^…^C7′ 3.782(2) Å), and mainly, by the sharing of perchlorate counteranions, which connect the polyaminic portion of ligands belonging to different columns.

### 2.6. Binding and Fluorescence Sensing of PFOA by [Zn***L1***]^2+^ and [Zn***L2***]^2+^

**Figure 11:**
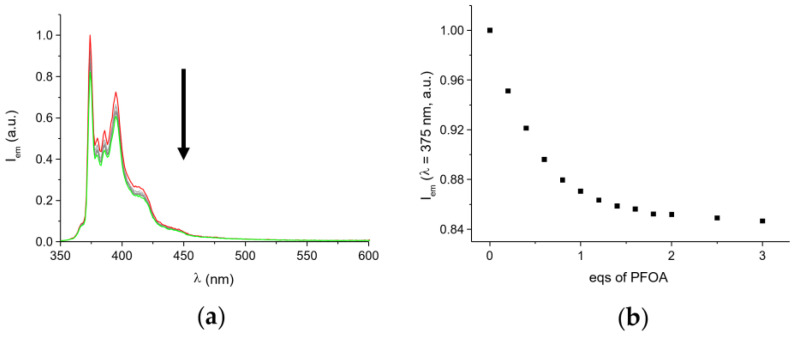
(**a**) Fluorescence emission spectra of [Zn**L1**]^2+^ at pH 8 (0.005 M TRIS buffer) in H_2_O/EtOH 50:50 (*v*/*v*) in the presence of increasing amounts of PFOA (spectra recorded in the absence and in the presence of 3 equivs. of PFOA are in red and green colors, respectively), [[Zn**L1**]^2+^] = 10^−^^5^ M, λ_exc_ = 340 nm) and (**b**) plot of the fluorescence emission of [Zn**L1**]^2+^ at 375 nm (arrow outlines the emission decrease as the equivs. of PFOA added increase from 0 to 3).

The presence of a coordinated Zn(II) cation, which features an unsaturated coordination sphere can be exploited in the coordination of PFOA, through the interaction of the anionic substrate (the carboxylic group only protonates in very acidic media, below pH 2) with the metal center. The binding features of the Zn(II) complexes of **L1** and **L2** and their possible use as optical probes were investigated through fluorescence emission measurements. Fluorescence titration experiments were carried out by adding a PFOA solution in H_2_O/EtOH (50:50 *v*/*v*) at pH 8 (0.005 M TRIS buffer) to a 10^−^^5^ M solution of the receptor in the same solvent. The increase in the concentration of PFOA induces a quenching of the pyrene fluorescent emission of both receptors, as shown in Figure 11 and Figure S33. The [Zn**L1**]^2+^ fluorescence emission decreases up to a [PFOA]/[Zn**L1**]^2+^ molar ratio of 1, to achieve an almost constant value for a [PFOA]/[Zn**L1**]^2+^ molar ratio greater than 1,4 suggesting the formation of 1:1 stoichiometry complexes. The quenching effect is only partial and can be ascribed to the weakening of the Zn-N bond upon PFOA binding, which can partially restore the PET effect from the amine groups to the fluorescent unit.

A similar result was also obtained for the Zn(II) complex of **L2**, although, in this case, the minimum of the fluorescence intensity is reached with a higher amount of PFOA (15 equivs.). Apparent constants of 3.2 and 3.4 log units for the addition of PFOA to the Zn(II) complexes with **L1** and **L2,** respectively, were calculated by data treatment with the Hypspec program, with detection limits (LODs) values 6.2 (**L1**)and 11.2 µM (**L2**). Both complexes show a binding ability lower than that found for the metal-free receptors at pH 4 and 7. However, as already observed for the polyammonium receptors, addition of PFOS to solutions of **L1** or **L2** at these pH values does not change the pyrene emission, indicating that, differently from PFOA, PFOS is not bound by the Zn(II) complexes. This result is in agreement with the known lower affinity for transition metal cations of the sulphonate group with respect to the carboxylate one.

Unfortunately, the ^1^H NMR spectra of the Zn(II) complexes with **L1** or **L2** show a remarkable line broadening in D_2_O/CD_3_OD (40:60 *v*/*v*) preventing their interpretation. The addition of PFOA or PFOS does not affect the fluxional character of the spectra. Therefore, ^1^H NMR experiments cannot be used to infer a hypothesis on the observed lower binding ability of the Zn(II) complexes with respect to the metal-free triamine at neutral or slightly acidic pH values. We also performed FT-IR spectra on the solid-state ternary complexes Zn**L1**(PFOA)Cl and Zn**L2**(PFOA)Cl obtained by slow evaporation of H_2_O/EtOH 50:50 (*v*/*v*) solutions containing **L1** or **L2**, ZnCl_2_^.^6H_2_O (1 equiv.) and PFOA (1 equiv.). The spectra show a slight, but not negligible (15 and 20 cm^−^^1^ for Zn**L1**(PFOA)Cl and Zn**L2**(PFOA)Cl, respectively) shift toward lower energy for the C=O stretching band, suggesting an interaction of the carboxylate group to the metal. Interestingly enough, the C-F stretching bands do not show significant shifts in the complex with respect to ‘free’ PFOA, suggesting that the perfluoroalkyl chain is not involved in complex stabilization. On the other hand, the crystal structure of the [Zn**L2**Cl]^+^ shows that Zn(II) complexation induces receptor stiffening which can prevent the interaction between pyrene and the perfluoroalkylchain of PFOA. Although the conclusion on structure in solution derived from X-ray structure can be misleading, we can tentatively suppose the carboxylate group replaces the chloride anion in the coordination sphere of the metal observed in the solid state structure shown in Figure 9. With the assumption, binding of the carboxylate group of PFOA to the metal would force the perfluoroalkyl chain to assume a spatial disposition far from the pyrene unit, preventing the formation of stabilizing hydrophobic interaction.

## 3. Materials and Methods

Compounds 1-(1-Methylpyrenyl)-1,4,7-triazaheptane (**L1**) and 1-(1-Methylpyrenyl)-1,5,9-triazanonane (**L2**) were prepared in accordance with the methods described in the literature [61]. NMR spectra were recorded on a Bruker 400 MHz (Bruker Corp.: Billerica, MA, USA) instrument. Reagents and solvents were from Sigma-Aldrich: Oakville, ON, USA).

### 3.1. Synthesis of 1-(1-Methylpyrenyl)-1,4,7-triazaheptane (***L1***)

Receptor **L1** was synthesized by modifying a previously reported procedure [61].

### 3.2. Synthesis of 1-(1-Methylpyrenyl)-1,5,9-triazanonane (***L2***)

A solution of bis(3-aminopropyl)amine (2.85 g, 21.7 mmol) in dry CH_2_Cl_2_ was added to a solution of pyrene-1-carbaldehyde (0.5 g, 2.17 mmol) in dry CH_2_Cl_2_. The mixture was refluxed with magnetic stirring for 3 h under a nitrogen atmosphere and then overnight at room temperature. The solution was recovered by filtration and concentrated by evaporation under a vacuum. The resulting oil was dissolved in dry ethanol (50 mL) and stirred with NaBH_4_ (0.246 g, 6.51 mmol) at 333 K for 2 h and at room temperature for 6 h. The resultant was concentrated by evaporation, dissolved in CH_2_Cl_2_, and washed with an aqueous NaOH solution (2 mol/L, 30 mL × 4). The organic layers were collected and dried over Na_2_SO_4_. After solvent removal under vacuum, a yellow oil was obtained. The product was dissolved in ethanol (20 mL) and HCl (37%) was dropwise added to the resulting solution, affording the triammonium chloride salt of **L2** (**L2**·3HCl) as a yellow solid. Yield 789 mg (80%). Anal. calcd. for C_23_H_30_Cl_3_N_3_: C 60.73, H 6.65, N 9.24. Found: C 59.66, H 6.21, N 8.83.

^1.^ H NMR (CDCl_3_ + 5% CD_3_OD, 400 MHz): δ (ppm) 8.28 (d, 1H, H6), 8.17–8.10 (m, 4H, H4-5-9-10), 8.02–7.93 (m, 4H, H2-3-7-8), 4.43 (s, 2H, 1_AL_), 2.78 (t, 2H, 2_AL_), 2.57–2.48 (m, 6H, 4-5-7_AL_), 1.70 (q, 2H, 3_AL_), 1.46 (q, 2H, 6_AL_). ^13^C NMR (DMSO-d_6_, 400 MHz): δ (ppm) 132.64, 131.66, 131.10, 130.31, 130.16, 129.57, 129.39, 128.35, 127.87, 127.04, 126.88, 125.92, 126.00, 124.94, 124.58, 123.97, 48.37, 45.35, 45.06, 44.97, 36.95, 24.49, 23.30. ESI MS (*m*/*z*) 346.22767 (z = 1, [**L2** + H]^+^).

### 3.3. Synthesis of [(Zn***L1***)(ClO_4_)_2_]

A solution of Zn(ClO_4_)_2_ (8.59 mg, 0.023 mmol) in MeOH (1 mL) was added to a solution of **L1** (10 mg, 0.023 mmol) in MeOH (5 mL). BuOH (1 mL) was added to the solution and the solvent was allowed to slowly evaporate. The obtained crystals were filtered and washed with BuOH. (7.6 mg, yield 57%). Anal calcd. for C_21_H_20_Cl_2_N_3_O_8_Zn: C 43.59, H 3.48, N 7.26. Found: C 44.03, H 3.87, N 7.65.

### 3.4. Synthesis of [(Zn***L2***)(ClO_4_)_2_]

A solution of Zn(ClO_4_)_2_ (8.19 mg, 0.022 mmol) in MeOH (1 mL) was added to a solution of **L2** (10 mg, 0.022 mmol) in MeOH (4 mL). BuOH (1 mL) was added to the solution and the solvent was allowed to slowly evaporate, to obtain crystal suitable for X-ray analysis, which were filtered off and washed with BuOH. (7.9 mg, yield 61%). Anal cald. for C_23_H_24_Cl_2_N_3_O_8_Zn: C 45.53, H 3.99, N 6.93. Found: C 46.61, H 3.37, N 5.84.

### 3.5. Electronic Absorption and Fluorescence Measurements

Absorption spectra were recorded on a Jasco V-670 spectrophotometer using ligand concentrations of 10**^−^**^5^ M. Emission spectra were recorded on a Horiba Scientific Fluoromax Plus, using an excitation wavelength of 340 nm and ligand concentrations of 10**^−^**^5^ M. All measurements were performed in a water/ethanol 1:1 (*v*/*v*) mixture at pH 7, using TRIS/HCl buffer 0.005 M, at 298.0 ± 0.1 K.

### 3.6. NMR Measurements

NMR titrations were carried out by using a Bruker Avance 400 MHz instrument, in D_2_O/CD_3_OD 40:60 (*v*/*v*) mixture at pH 7, by addition of PFOA to 0.001 M solutions of **L1** or **L2**. The pH was adjusted at 7 by adding small amounts of NaOD or DCl after each addition of PFOA to solutions of **L1** or **L2**.

### 3.7. Potentiometric Measurements

Potentiometric (pH-metric) titrations, employed to determine ligand protonation and metal complexation constants, were performed in H_2_O/EtOH 50:50 *v*/*v*, with 0.1 M NMe_4_Cl as ionic strength, at 298.1 ± 0.1 K using an automated apparatus and a procedure previously described [75]. Acquisition of the emf data was performed with the computer program PASAT [76] The combined Metrohm 6.0262.100 electrode was calibrated as a hydrogen-ion concentration probe by titration of previously standardized amounts of HCl solutions with CO_2_-free NMe_4_OH solutions and determining the equivalent point by Gran’s method [77], which gives the standard potential, E°, and the ionic product of water (experimental p*K*_w_ = 14.52(1) in above stated conditions). The computer program HYPERQUAD [66] was used to calculate the protonation constants of the receptors and substrates and the stability constants of the adducts from the potentiometric data. The concentration of ligands was about 5 × 10^−4^ M in all experiments while the concentration of Zn(II) was 4 × 10^−3^ M. The studied pH range was 2.5–10 for **L1** and **L2**, for the determination of ligand protonation constants, and 2.5–8-5 for **L1** and 2.5–7.5 for **L2**, in the case of the determination of the metal complexation constants. Precipitation of the Zn(II) complex of **L1** and **L2** is observed at pH > 8.5 and pH > 7.5, respectively. No receptor-anion interaction was observed for PFOS (up to 5 equivs.). Reliable determination of PFOA binding constants with the protonated forms of **L1** and **L2** was prevented by solubility issues.

### 3.8. Limit of Detection (LOD) Determination

The detection limit was determined from the fluorescence titrations data based on a reported method [78]. Six measurements of the **L1**, **L2**, [Zn**L1**]^2+^, and [Zn**L2**]^2+^ fluorescence spectra at pH 4 and 7 in H_2_O/EtOH 50:50 (*v*/*v*) were made, and the standard deviation of the blank measurements was calculated. The fluorescence intensity I_0_/I at 375 nm (I_0_: fluorescence intensity of the blank at 375 nm; I: fluorescence intensity of the sample at 375 nm) in the presence of increasing amount of PFOA was plotted as a concentration of PFOA and gave a straight line, whose slope (K) was calculated. The detection limit was calculated by using the equation:

The detection limit (LOD) = 3s/K,(1)

where s is the standard deviation of blank measurement and K is the slope between the fluorescence versus PFOA concentration.

### 3.9. FT-IR Measurements

FT-IR measurements were performed on freshly prepared complexes obtained by adding ZnCl_2_^.^6H_2_O (1 equiv.) and PFOA (1 equiv.) to a solution of the ligand **L1** or **L2** in an H_2_O/EtOH 50:50 (*v*/*v*) mixture. The pH of the solution was adjusted at pH 8 by the addition of a small amount of a 0.1M solution of NaOH in an H_2_O/EtOH 50:50 (*v*/*v*) mixture. Solid complexes were isolated by slow evaporation of the resulting solution. The spectra were recorded with an IRAffinity-1S Shimadzu instrument.

Anal calcd. for Zn**L1**(PFOA)Cl, C_29_H_20_ClF_15_N_3_O_2_Zn: C 42.05, H 2.43, N 5.07. Found: C 41.23, H 3.57, N 6.34. Anal calcd. for Zn**L2**(PFOA)Cl, C_31_H_24_ClF_15_N_3_O_2_Zn: C 43.48, H 2.82, N 4.91. Found: C 44.76, H 3.54, N 4.21.

### 3.10. Single-Crystal X-ray Diffraction

Single crystal X-ray diffraction data of 6 were collected on a Bruker Apex-II diffractometer equipped with a CCD detector (T = 100 K, Cu–Kα radiation (λ = 1.54184 Å). Data were collected with the APEX2 software, [2] while data integration and reduction were performed with the Bruker SAINT software. [3] The crystal structure was solved using the SIR-2004 package [79] and refined by full-matrix least-squares against *F^2^* using all data (SHELXL-2018/3) [80]. All the non-hydrogen atoms were refined with anisotropic displacement parameters, while all the hydrogen atoms were found in the Fourier Density Maps, their coordinates were freely refined while their thermal parameter was set in accordance with that of the atoms to which they are bonded. Geometrical calculations were performed by PARST97 [81] and molecular plots were produced by the program CCDC Mercury [82]. Crystallographic data and refinement parameters are reported in Table S2. Crystal Data for [Zn(C_23_H_25_N_3_)Cl]ClO_4_ (M = 543.73 g/mol): triclinic, space group P-1 (no. 1), a = 8.7070(2) Å, b = 11.4358(2) Å, c = 12.7837(3) Å, α = 77.662(1)°, β = 93.3735(9)°, γ = 68.582(1)°, V = 1157.50(4) Å^3^, Z = 2, T = 100 K, μ(CuKα) = 3.903 mm^−1^, Dcalc = 1.560 mg/cm^3^, 33,464 reflections measured (3.539° ≤ Θ ≤ 72.429°), 4558 unique (Rint = 0.0448) which were used in all calculations. The final R indices [I > 2**σ**] are 0.0281/0.0803, R indices (all data) are 0.0305/0.0834 and the GoF is 0.884. CCDC 2237493 contains the supplementary crystallographic data for this paper. These data can be obtained free of charge via http://www.ccdc.cam.ac.uk/conts/retrieving.html (or from the CCDC, 12 Union Road, Cambridge CB2 1EZ, UK; Fax: +44-1223-336033; E-mail: deposit@ccdc.cam.ac.uk).

## 4. Conclusions

These results point out that the straightforward coupling of a polar hydrophilic section is able to interact via charge-charge and H-bonding with the negatively charged perfluoroalkyl carboxylate groups, with a large aromatic unit, which can give hydrophobic interactions with the perfluorinated aliphatic chain may result in molecular receptors able to form stable complexes with PFOA, with great selectivity for this analyte over PFOS, whose sulphonate group shows a poor tendency to give H-bonding. This is the case of the triamine receptors **L1** and **L2**, that, at neutral or slightly acidic pH values, form protonated species. Despite their simple structures and their straightforward synthetic accessibility, they can form complexes with PFOA featuring stability higher than those formed by most of the previously reported receptors and, at the same time, selectivity for PFOA over PFOS. The ability of triamine to form complexes with Zn(II), a metal ion known for its ability to change/expand its coordination sphere in order to bind exogenous species represents an ‘added value’ of these molecular receptors. In fact, this metal ion can be exploited as an anchoring point for the carboxylate group of PFOA, a structural characteristic that leads to the formation of stable complexes between the Zn(II) complexes with **L1** and **L2** and PFOA and selectivity for the latter over PFOS. The Zn(II) complexes with PFOA, however, are less stable than that formed by the protonated species of the receptors, likely due to the rigidity of molecular architecture of the Zn(II) complexes with the present receptors, well outlined by the crystal structure of the [Zn**L2**Cl]^+^ cation. As a result, binding of the carboxylate of PFOA to Zn(II) can force the perfluoroalkyl chain to stay far from the pyrene unit, preventing the formation of stabilizing hydrophobic interactions with this aromatic moiety.

Pyrene can be exploited not only to bind PFOA in cooperation with the polyamine aliphatic chain (when protonated or bound to Zn(II)), but also to signal the binding event through changes in its emission properties. Indeed, the binding of PFOA induces fluorescence quenching, more marked in the case of the metal-free receptors. Although the limit for PFOA detection is not particularly exceptional, PFOA is selectively detected over PFOS by both receptors, a peculiar characteristic in the panorama of molecular optical sensors for these analytes. Furthermore, the performance of the chemosensors in PFOA detection can be, in perspective, enhanced by their incorporation in nanostructured materials, in which energy transfer cooperative effects may occur between closely placed fluorescent probes. At the same time, the simple synthetic procedure used can be exploited to develop different chemosensors, differing from **L1** and **L2** in the polyamine chain and/or in the fluorogenic moiety used, in order to develop a library of fluorescent probes. From this point of view, the assembly of arrays of chemosensors may be exploited to optimize the selectivity of the recognition process.

The above considerations outline that coupling a polyamine chain with a nearby fluorescent hydrophobic moiety may represent a novel approach for the development of new optical PFOA chemosensors, simultaneously displaying high stability of the adducts, fluorescence response and particular selectivity properties. Altogether, these characteristics make fluorescent polyamine-based receptors a promising tool for the binding and detection of this environmental contaminant.

## Figures and Tables

**Table 1:** Protonation constants of **L1** and **L2** in H_2_O/EtOH (50:50 *v*/*v*) (0.1 M NMe_4_Cl, 298.1 ± 0.1 K).

Equilibrium	L1	L2
L + H^+^ = HL^+^	9.44(2)	9.97(2)
HL^+^ + H^+^ = H_2_L^2+^	7.67(1)	8.38(1)
H_2_L^2+^ + H^+^ = H_3_L^3+^	3.64(2)	6.78(2)

**Table 2:** Zn(II) binding constants of **L1** and **L2** in H_2_O/EtOH (50:50 *v*/*v*) (0.1 M NMe_4_Cl, 298.1 ± 0.1 K).

Equilibrium	L1	L2
L + Zn^2+^ = ZnL^+^	8.69(3)	7.88(2)
ZnL^+^ + OH^−^ = ZnLOH^+^	-	5.92(2)

## Data Availability

The data supporting the findings of this study are available within the article and its Supplementary Materials.

## Supplementary Materials

The following supporting information can be downloaded at: https://www.mdpi.com/article/10.3390/molecules28114552/s1, Figure S1: ^1^H NMR spectrum of compound **L1** in CD_3_OD/D_2_O; Figure S2: ^1^H NMR spectrum of compound **L1** in DMSO-d6; Figure S3: ^1^H-^1^H -COSY NMR spectrum of compound **L1**; Figure S4: ^13^C NMR spectrum of compound **L1**; Figure S5: HR-ESI MS of compound **L1**; Figure S6: Isotopic pattern of the [**L1** + H]^+^ (z = 1) ion; Figure S7: ^1^H NMR spectrum of compound **L2** in CDCl_3_/CD_3_OD; Figure S8: ^1^H NMR spectrum of compound **L2** in DMSO-d6; Figure S9: ^1^H-^1^H -COSY NMR spectrum of compound **L2**; Figure S10: ^13^C NMR spectrum of compound **L2**; Figure S11:HR-ESI MS of compound **L2**; Figure S12: Isotopic pattern of the [**L2** + H]^+^ (z = 1) ion; Figure S13: Absorption spectra of **L1** and **L2** at different pH values; Figure S14: Absorption spectra of **L1** at pH 7 in the presence of increasing amount of PFOA; Figure S15: Absorption and emission spectra of **L2** at pH 7 in the presence of increasing amount of PFOA; Figure S16: Emission spectra of **L1** and **L2** at pH 7 in the presence of increasing amount of PFOS; Figure S17: Absorption spectra of **L1** at pH 4 in the presence of increasing amount of PFOA; Figure S18: Absorption and emission spectra of **L2** at pH 4 in the presence of increasing amount of PFOA; Figure S19: Emission spectra of **L1** and **L2** at pH 4 in the presence of increasing amount of PFOS; Figure S20: Emission spectra of **L1** and **L2** at pH 4 in the presence of increasing amount of PFOA at 308 K; Figure S21: Emission spectra of **L1** and **L2** at pH 4 in the presence of increasing amount of PFOA at 288 K; Figure S22: Plot of fluorescence emission I_0_/I of **L1** and **L2** at pH 7 in the presence of increasing amount of PFOA; Figure S23: Plot of fluorescence emission I_0_/I of **L1** and **L2** at pH 4 in the presence of increasing amount of PFOA; Figure S24: ^1^H NMR spectra of **L1** at pH 4 in the presence of increasing amounts of PFOS; Figure S25: ^1^H NMR spectra of **L1** at pH 7 in the presence of increasing amounts of PFOA; Figure S26: ^1^H NMR spectra of **L2** at pH 7 in the presence of increasing amounts of PFOA; Figure S27: ^19^F NMR spectra of PFOA at pH 7 in the presence of increasing amounts of **L1**; Figure S28: Emission spectra of **L1** and **L2** at pH 4 in the presence of different interfering agents; Figure S29: Emission spectra of **L1** and **L2** at pH 4 in the presence of 100 equivs. of PFOA and 200 equivs. of interfering agent; Figure S30: Absorption spectra of **L1** in the presence of increasing amount of Zn(II); Figure S31: Absorption and emission spectra of **L2** in the presence of increasing amount of Zn(II); Figure S32: Absorption spectra of [Zn**L1**]^2+^ in the presence of increasing amount of PFOA; Figure S33: Absorption and emission spectra of [Zn**L2**]^2+^ in the presence of increasing amount of PFOA; Figure S34: Plot of fluorescence emission I_0_/I of [Zn**L1**]^2+^ and [Zn**L2**]^2+^ in the presence of increasing amount of PFOA; Figure S35: FT-IR solid state spectra of PFOA sodium salt and the Zn**L1**(PFOA)Cl complex; Figure S36. FT-IR solid state spectra of PFOA sodium salt and the Zn**L2**(PFOA)Cl complex. Figure S37: Emission spectra of [Zn**L1**]**^2+^** and [Zn**L2**]^2+^ at pH 8 in the presence of the increasing amount of PFOS; Table S1: Bond distances and angles defining the Zn(II) coordination environment in the crystal structure of **6**; Table S2: Crystallographic data and refinement parameters for **6**. Supplementary crystallographic data for this paper have been deposited at the Cambridge Crystallographic Data Centre (CCDC) with deposition number 2237493 ([Zn**L2**Cl](ClO_4_)).

## Sample Availability

Samples of the compounds **L1** and **L2** are available from the authors.
