# Supplementary material for: Pyrene-Containing Polyamines as Fluorescent Receptors for Recognition of PFOA in Aqueous Media
Source: Molecules. 2023 Jun 5;28(11):4552. doi: 10.3390/molecules28114552 (PMC10254721; doi:10.3390/molecules28114552)
Supplement: Supplementary file 1 [file molecules-28-04552-s001.zip › molecules-2407239-supplementary.pdf]

Pyrene containing polyamines as fluorescent receptors for the recognition of PFOA in aqueous media

Yschtar Tecla Simonini Steiner, Giammarco Maria Romano, Matteo Savastano, Lara Massai, Martina Lippi, Paola Paoli, Patrizia Rossi and Andrea Bencini

Supplementary Materials (SM)

Contents

Synthesis of 1-(1-Methylpyrenyl)-1,4,7-triazaheptane (L1)……………………………………………3
Figure S1. ^{1}H NMR spectrum of compound **L1** in CD_3OD/D_2O……………………………3
Figure S2. ^{1}H NMR spectrum of compound **L1** in DMSO-d6…………………………………4
Figure S3. ^{1}H-^{1}H -COSY NMR spectrum of compound **L1**…………………………………….5
Figure S4. ^{13}C NMR spectrum of compound **L1**………………………………………………5
Figure S5. HR-ESI MS of compound **L1**……………………………………………………….6
Figure S6. Isotopic pattern of the [**L1**+H]$^+$ (z = 1) ion……………………………………………...6
Figure S7. ^{1}H NMR spectrum of compound **L2** in $CDCl_3/CD_3OD$..…...………………………7
Figure S8. ^{1}H NMR spectrum of compound **L2** in DMSO-d6…………………………………8
Figure S9. ^{1}H-^{1}H -COSY NMR spectrum of compound **L2**……………………………………9
Figure S10. ^{13}C NMR spectrum of compound **L2**…….…………………………………………10
Figure S11. HR-ESI MS of compound **L2**……………………………………..……………...11
Figure S12. Isotopic pattern of the [**L2**+H]$^+$ (z = 1) ion………………………………………...11
Figure S13. Absorption spectra of **L1** and **L2** at different pH values………………………..12
Figure S14. Absorption spectra of **L1** at pH 7 in the presence of increasing amount of PFOA……………………………………………………………………………………12
Figure S15. Absorption and emission spectra of **L2** at pH 7 in the presence of increasing amount of PFOA…………………………………………………………………………..13
Figure S16. Emission spectra of **L1** and **L2** at pH 7 in the presence of increasing amount of PFOS…………………………………………………………………………………..13
Figure S17. Absorption spectra of **L1** at pH 4 in the presence of increasing amount of PFOA……………………………………………………………………………………14
Figure S18. Absorption and emission spectra of **L2** at pH 4 in the presence of increasing amount of PFOA…………………………………………………………………………..14
Figure S19. Emission spectra of **L1** and **L2** at pH 4 in the presence of increasing amount of PFOS…………………………………………………………………………………..15

Figure S20: Emission spectra of **L1** and **L2** at pH 4 in the presence of increasing amount of PFOA at 35°C308 K……………………………………………………………………………..15
Figure S21: Emission spectra of **L1** and **L2** at pH 4 in the presence of increasing amount of PFOA at 15°C288 K……………………………………………………………………………..15
Figure S22. Plot of fluorescence emission I_0/I of **L1** and **L2** at pH 7 in the presence of increasing amount of PFOA……………………………………………………………………...16
Figure S23. Plot of fluorescence emission I_0/I of **L1** and **L2** at pH 4 in the presence of increasing amount of PFOA……………………………………………………………………...16
Figure S24. ^{1}H NMR spectra of **L1** at pH 4 in the presence of increasing amounts of PFOS………………………………………………………………………………………………..17
Figure S25. ^{1}H NMR spectra of **L1** at pH 7 in the presence of increasing amounts of PFOA……………………………………………………………………………………………….18
Figure S26. ^{1}H NMR spectra of **L2** at pH 7 in the presence of increasing amounts of PFOA……………………………………………………………………………………………….19
Figure S27. ^{19}F NMR spectra of PFOA at pH 7 in the presence of increasing amounts of **L1**………………………………………………………………………………………………....19
Figure S28: Emission spectra of **L1** and **L2** at pH 4 in the presence of different interfering agents. …………………………...……………………………………………………………....20
Figure S29: Emission spectra of **L1** and **L2** at pH 4 in the presence of 100 equivs. of PFOA and 200 equivs. of interfering agent…………………………………….………………....21
Figure S30. Absorption spectra of **L1** in the presence of increasing amount of Zn(II)…….21
Figure S31. Absorption and emission spectra of **L2** in the presence of increasing amount of Zn(II)……………………………………………………………………………………………..22
Figure S32. Absorption spectra of [Zn**L1**]$^{2+}$ in the presence of increasing amount of PFOA……………………………………………………………………………………………….22
Figure S33. Absorption and emission spectra of [Zn**L2**]$^{2+}$ in the presence of increasing amount of PFOA……………………………………………………………………………..23
Figure S34. Plot of fluorescence emission I_0/I of [Zn**L1**]$^{2+}$ and [Zn**L2**]$^{2+}$ in the presence of increasing amount of PFOA……………………………………………………………………...23
Figure S35: FT-IR solid state spectra of PFOA sodium salt and the Zn**L1**(PFOA)Cl complex.………………………………………………………………………….................24
Figure S36: FT-IR solid state spectra of PFOA sodium salt and the Zn**L2**(PFOA)Cl complex……………………………………………………………………………….…...25
Figure S37. Emission spectra of [Zn**L1**]$^{2+}$ and [Zn**L2**]$^{2+}$ at pH 8 in the presence of increasing amount of PFOS……………………………………………………………………...25
Table S1. Bond distances and angles defining the Zn(II) coordination environment in the crystal structure of **6**………………………………………………………………………25
Table S2. Crystallographic data and refinement parameters for **6**…………………………..…25

Synthesis of 1-(1-Methylpyrenyl)-1,4,7-triazaheptane (L1).

A solution of diethylenetriamine (2.23 g, 21.7 mmol) in dry CH_2Cl_2 was added to a solution of pyrene-1-carbaldehyde (0.5 g, 2.17 mmol) in dry CH_2Cl_2. The mixture was refluxed with magnetic stirring for 3 h under nitrogen atmosphere and then overnight at room temperature. The solution was recovered by filtration and concentrated by evaporation under vacuum. The resulting oil was dissolved in dry ethanol (50 mL) and stirred with $NaBH_4$ (0.246 g, 6.51 mmol) at 333 K for 2 h and at room temperature for 6 h. The resultant was concentrated by evaporation, dissolved in CH_2Cl_2, washed with an aqueous NaOH solution (2 mol/L, 30 mL × 4). The organic layers were collected and dried over Na_2SO_4. After solvent removal under vacuum a yellow oil was obtained. The product was dissolved in ethanol (20 mL) and HCl (37%,) was dropwise added to the resulting solution, affording the tris-hydrochloride salt of **L1** (**L1**·3HCl) as a yellow solid. Yield 768 mg (85%). Anal. cald. for $C_{21}H_{26}Cl_3N_3$: C 59.10, H 6.14, N 9.85. Found: C 58.78, H 6.46, N 9.13. ^1H NMR (CD_3OD + 30% D_2O, 400 MHz): δ(ppm) 8.30-8.22 (m, 5H, H4-5-6-9-10), 8.16-8.07 (m, 4H, H2-3-7-8), 3.26 (t, 2H, 2_{AL}), 3.08 (t, 2H, 4_{AL}), 2.98 (t, 2H, 3_{AL}), 2.91 (t, 2H, 5_{AL}). ^{13}C NMR (DMSO-d_6, 400 MHz): δ(ppm) 131.89, 131.20, 130.70, 129.68, 129.53, 128.69, 127.7, 127.08, 126.29, 126.14, 125.81, 125.29, 124.38, 124.13, 123.87. ESI MS (m/z) 318.19571 (z = 1, [**L1** + H]$^+$).

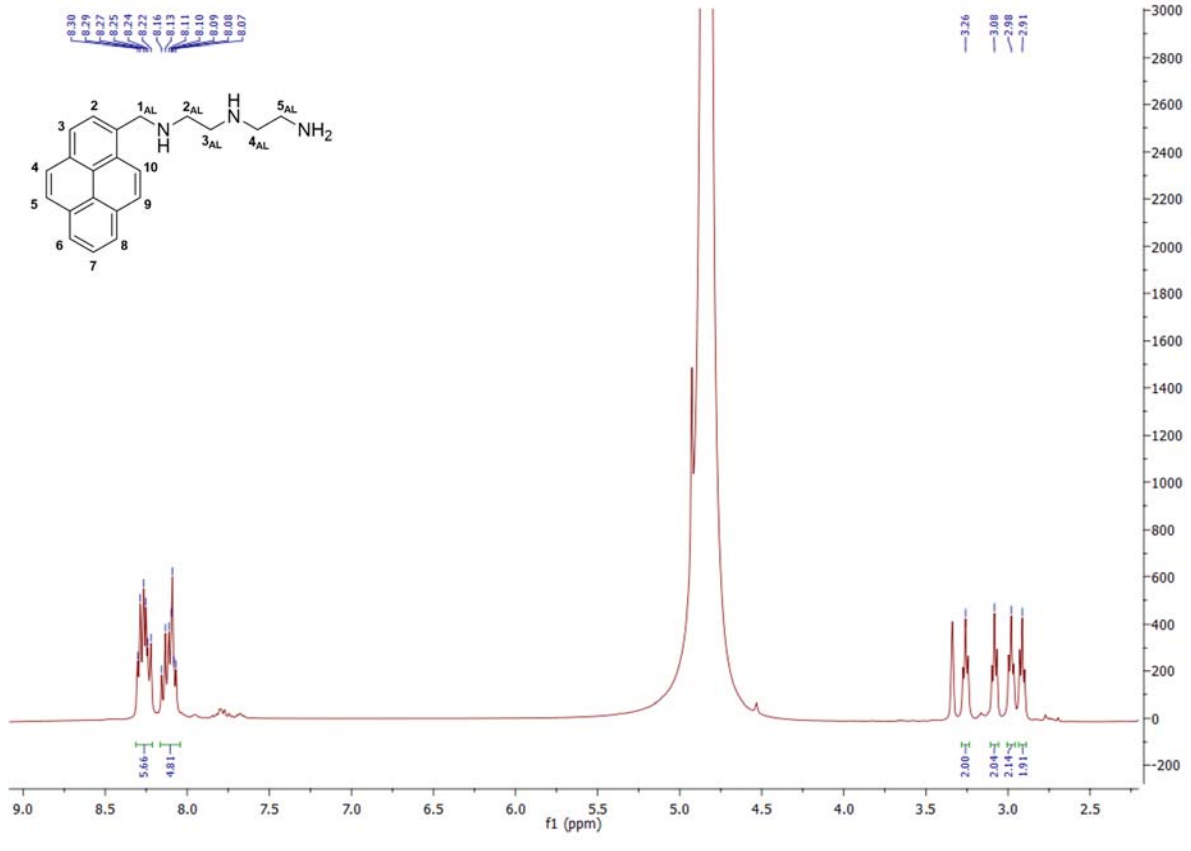

Figure S1. ^1H NMR spectrum of compound **L1** (CD_3OD + 30% D_2O, 400 MHz): δ(ppm) 8.30-8.22 (m, 5H, H4-5-6-9-10), 8.16-8.07 (m, 4H, H2-3-7-8), 3.26 (t, 2H, 2_{AL}), 3.08 (t, 2H, 4_{AL}), 2.98 (t, 2H, 3_{AL}), 2.91 (t, 2H, 5_{AL}).

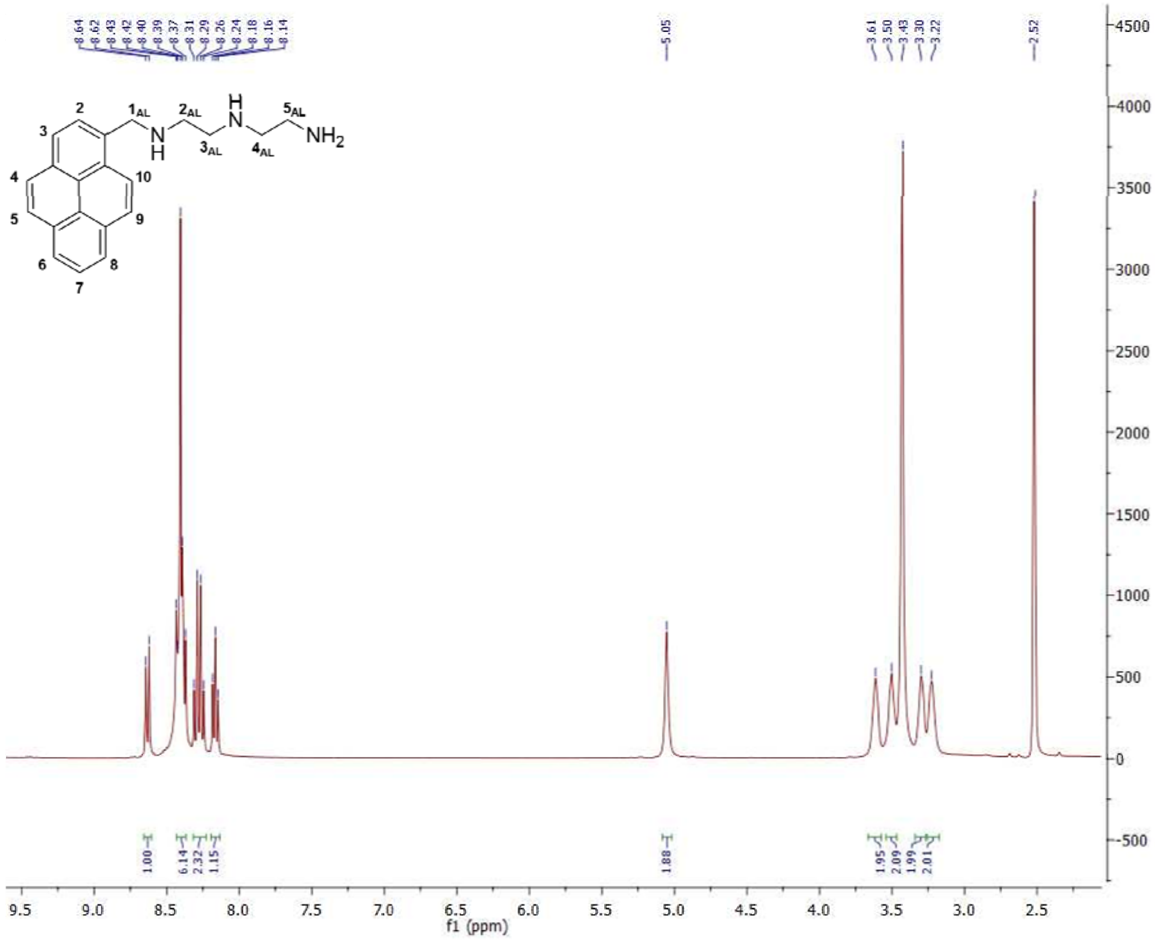

Figure S2. ^{1}H NMR spectrum of compound **L1** (DMSO-d6, 400 MHz): δ(ppm) 8.63 (1H, d), 8.37-8.43 (5H, m), 8.31-8.24 (2H, m), 8.16 (1H, t), 5.05 (2H, s), 3.61 (2H, broad singlet), 3.50 (2H, broad singlet), 3.30 (2H, br s), 3.22 (2H, broad singlet).

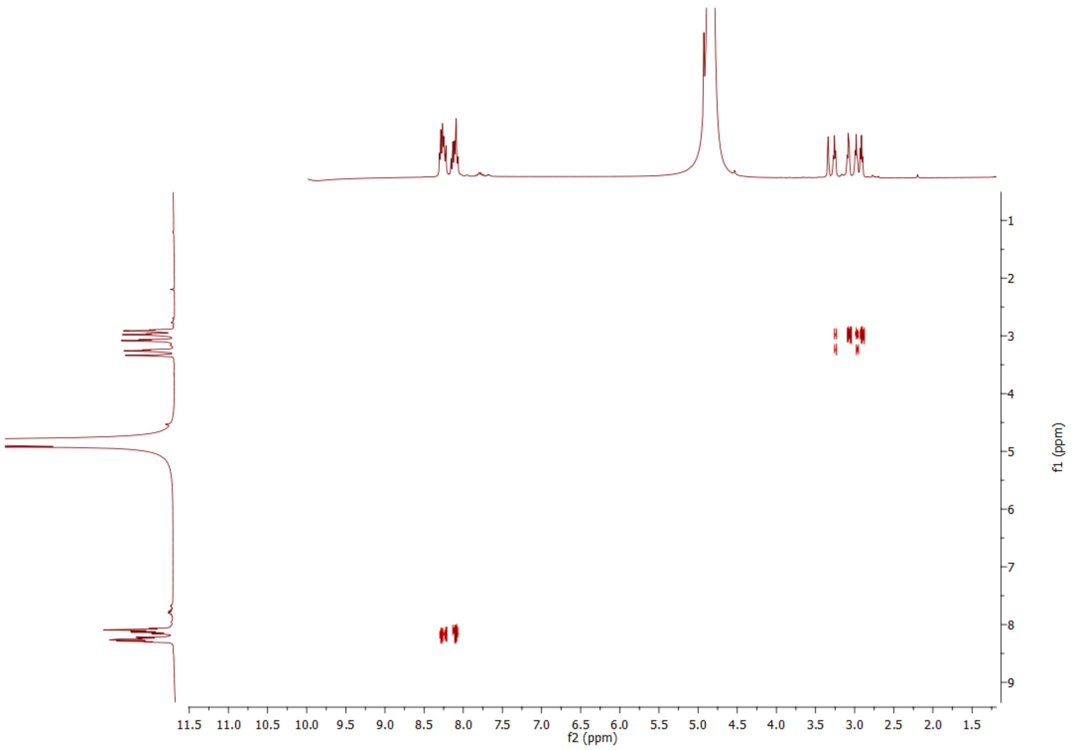

Figure S3. ^{1}H-^{1}H -COSY NMR spectrum of compound **L1** (CD_3OD + 30% D_2O-d6, 400 MHz).

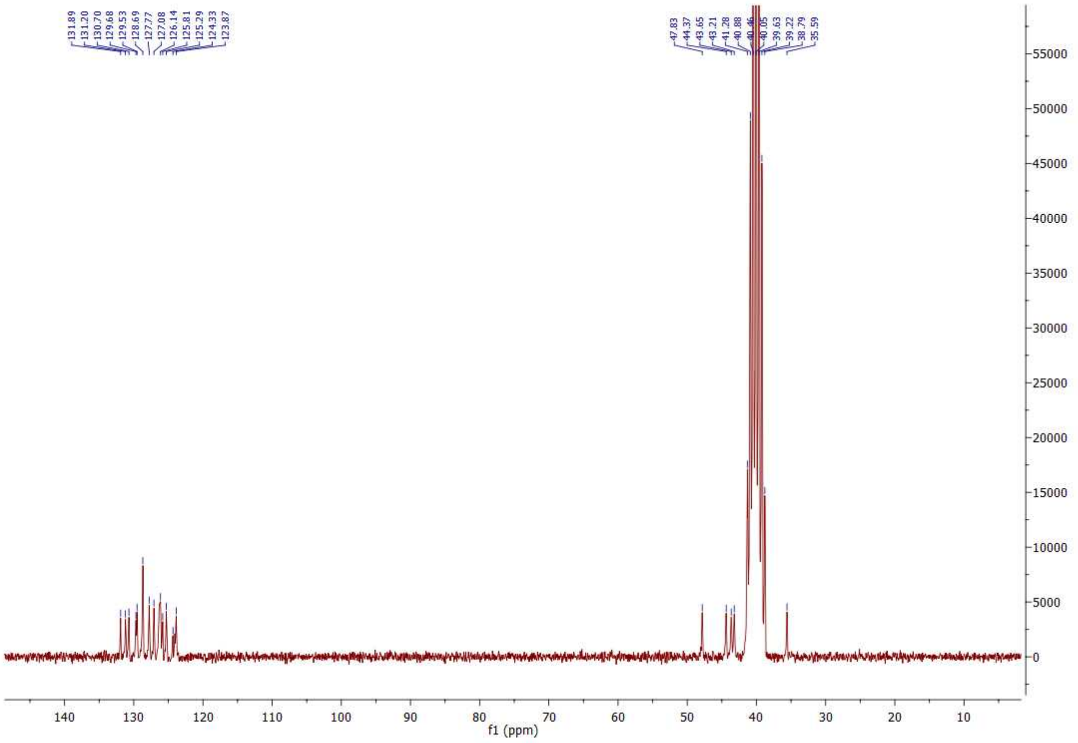

Figure S4. ^{13}C NMR spectrum of compound **L1** (DMSO-d6, 200 MHz): δ(ppm) 131.89, 131.20, 130.70, 129.68, 129.53, 128.69, 127.77, 127.08, 126.14, 125.81, 125.29, 124.33, 123.87, 47.83, 44.37, 43.65, 43.21, 35.59.

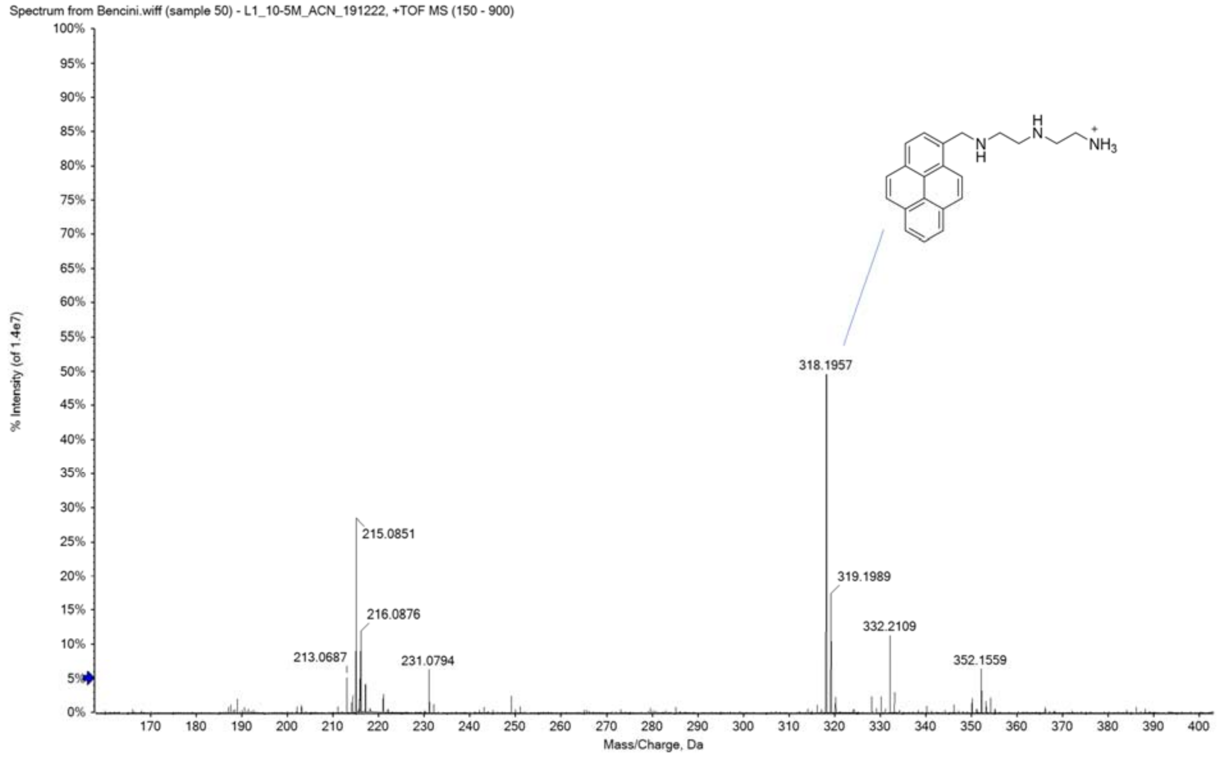

Figure S5. High resolution mass spectrum of compound **L1** in CH_3CN; 318.1957 (z = 1, [**L1** + H]$^+$).

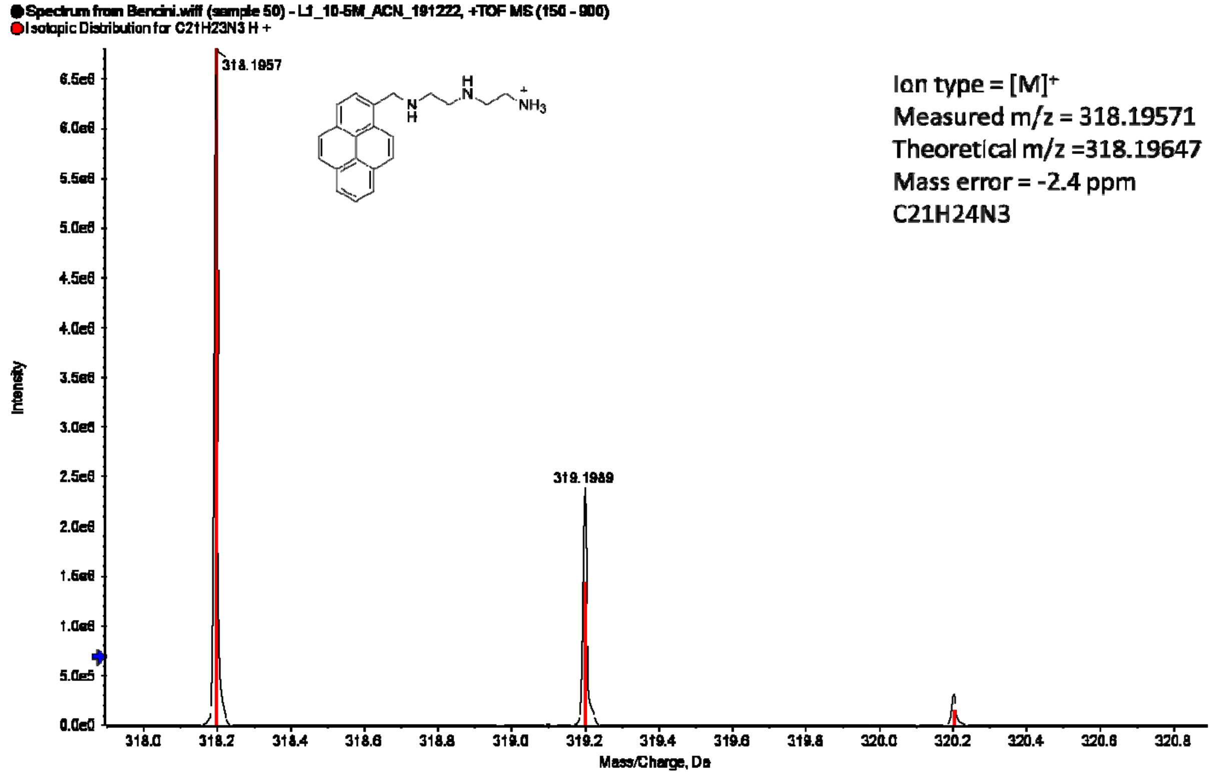

Figure S6. Isotopic pattern of the [**L1**+H]$^+$ (z = 1) ion, with measured (black) and theoretical (red) m/z value of the most abundant isotopic peak, 317.9-320.9 Da region.

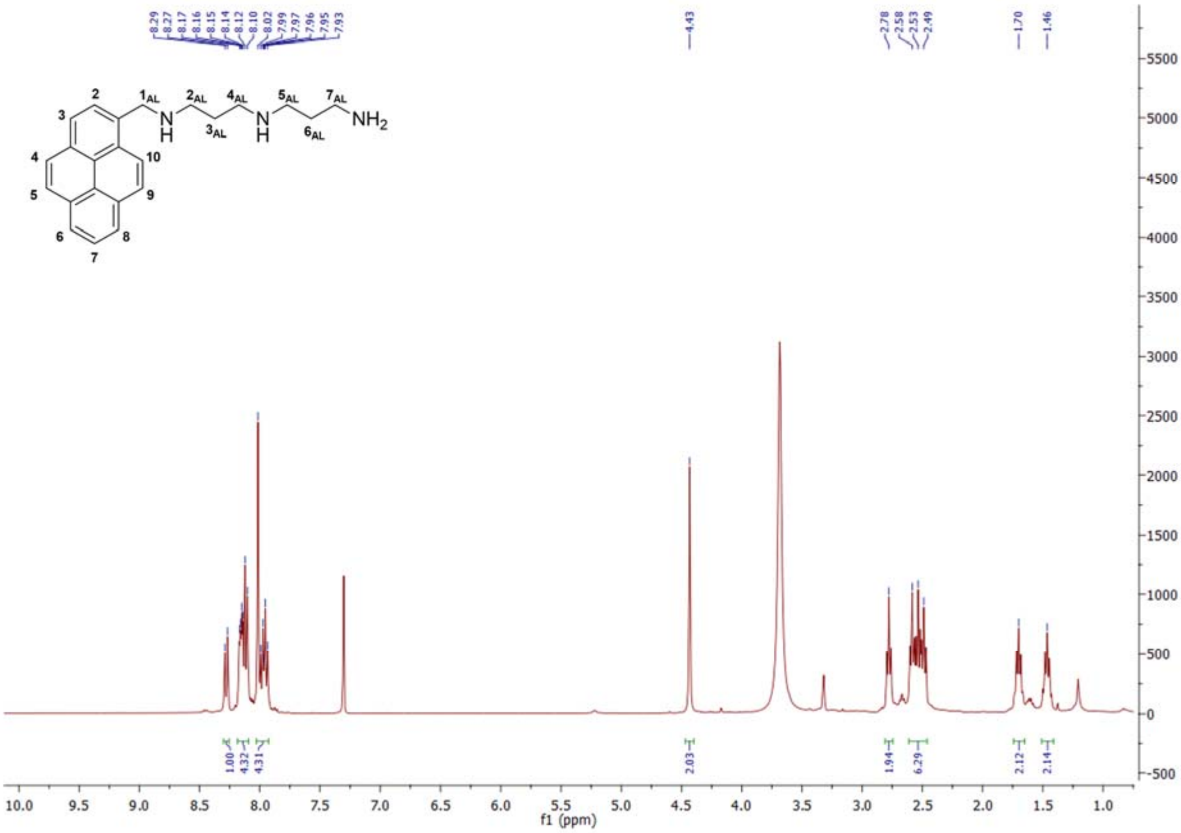

Figure S7. ^{1}H NMR spectrum of compound **L2** ($CDCl_3$ + 5% CD_3OD, 400 MHz): δ(ppm) 8.28 (d, 1H, H6), 8.17-8.10 (m, 4H, H4-5-9-10), 8.02-7.93 (m, 4H, H2-3-7-8), 4.43 (s, 2H, 1AL), 2.78 (t, 2H, 2AL), 2.57-2.48 (m, 6H, 4-5-7AL), 1.70 (q, 2H, 3AL), 1.46 (q, 2H, 6AL).

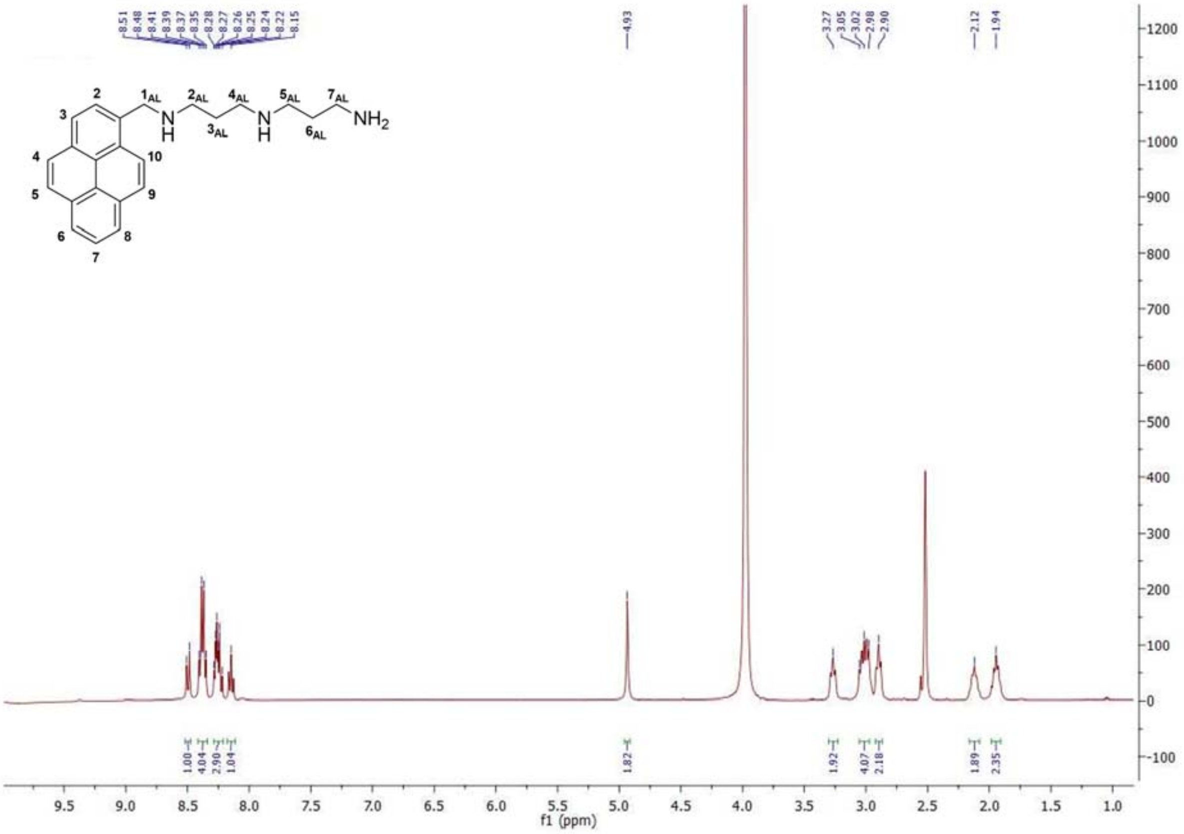

Figure S8. ^{1}H NMR spectrum of compound **L2** (DMSO-d$_6$, 400 MHz): δ(ppm) 8.50 (1H, d), 8.41-8.35 (4H, m), 8.28-8.22 (3H, m), 8.15 (1H, t), 4.93 (2H, s), 3.27 (2H, t), 3.05-2.98 (4H, m), 2.90 (2H, t), 2.12 (2H, quintet), 1.94 (2H, quintet).

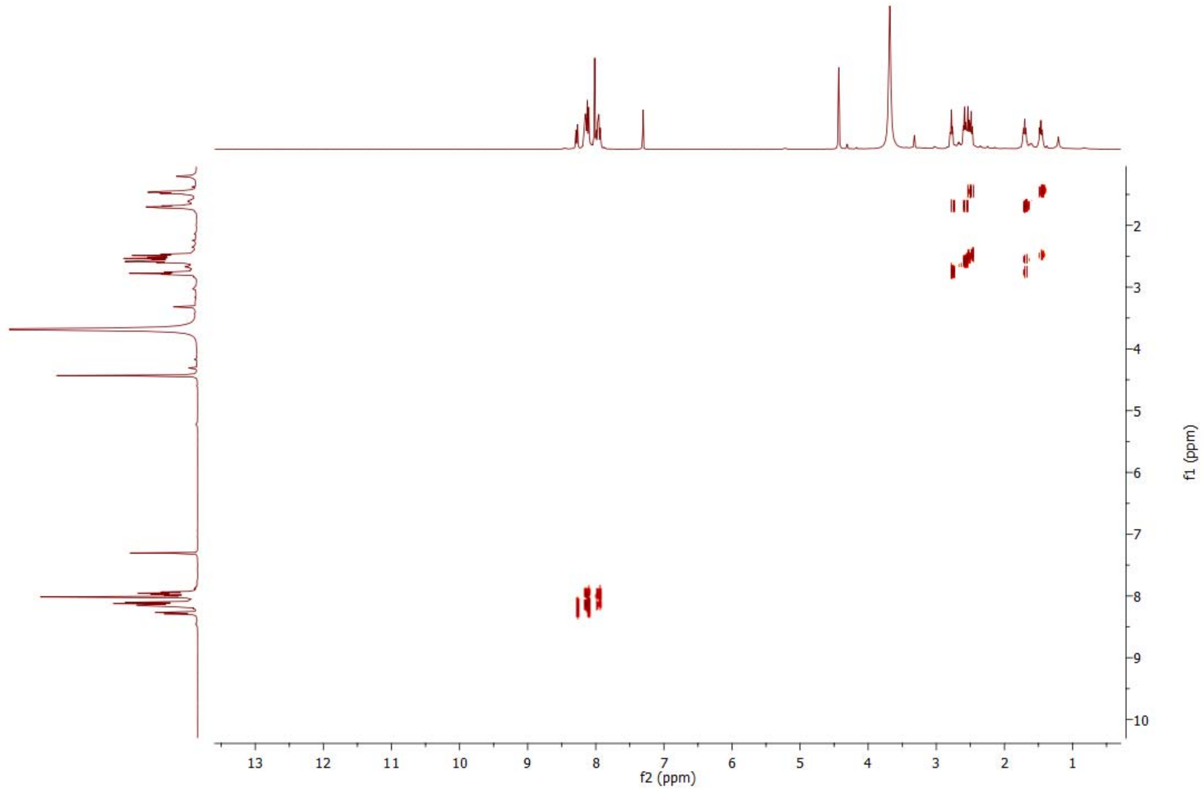

Figure S9. ^1H-^1H -COSY NMR spectrum of compound **L2** ($CDCl_3$ + 5% CD_3OD, 400 MHz).

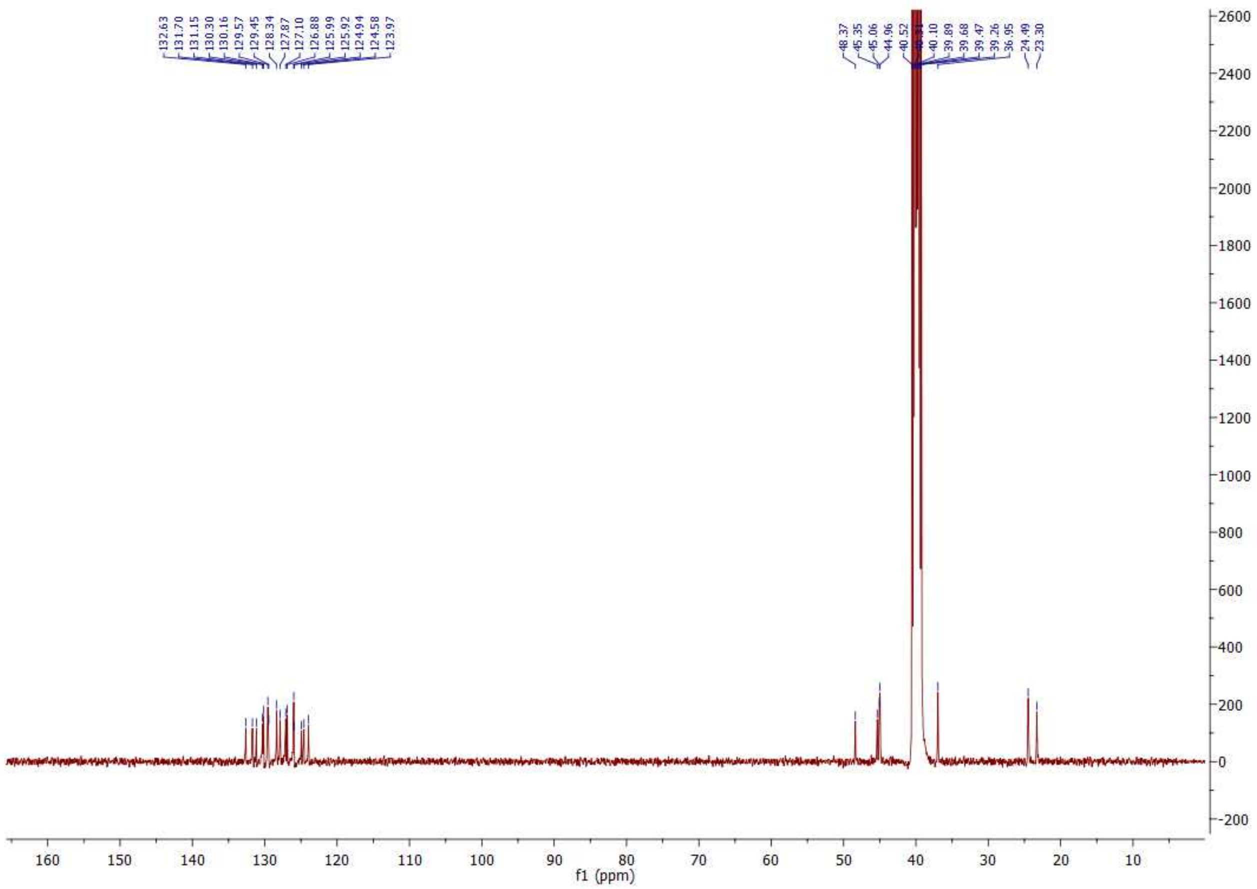

Figure S10. ^{13}C NMR spectrum of compound **L2** (DMSO-d6, 200 MHz): δ(ppm) 132.64, 131.66, 131.10, 130.31, 130.16, 129.57, 129.39, 128.35, 127.87, 127.04, 126.88, 125.92, 126.00, 124.94, 124.58, 123.97, 48.37, 45.35, 45.06, 44.97, 36.95, 24.49, 23.30.

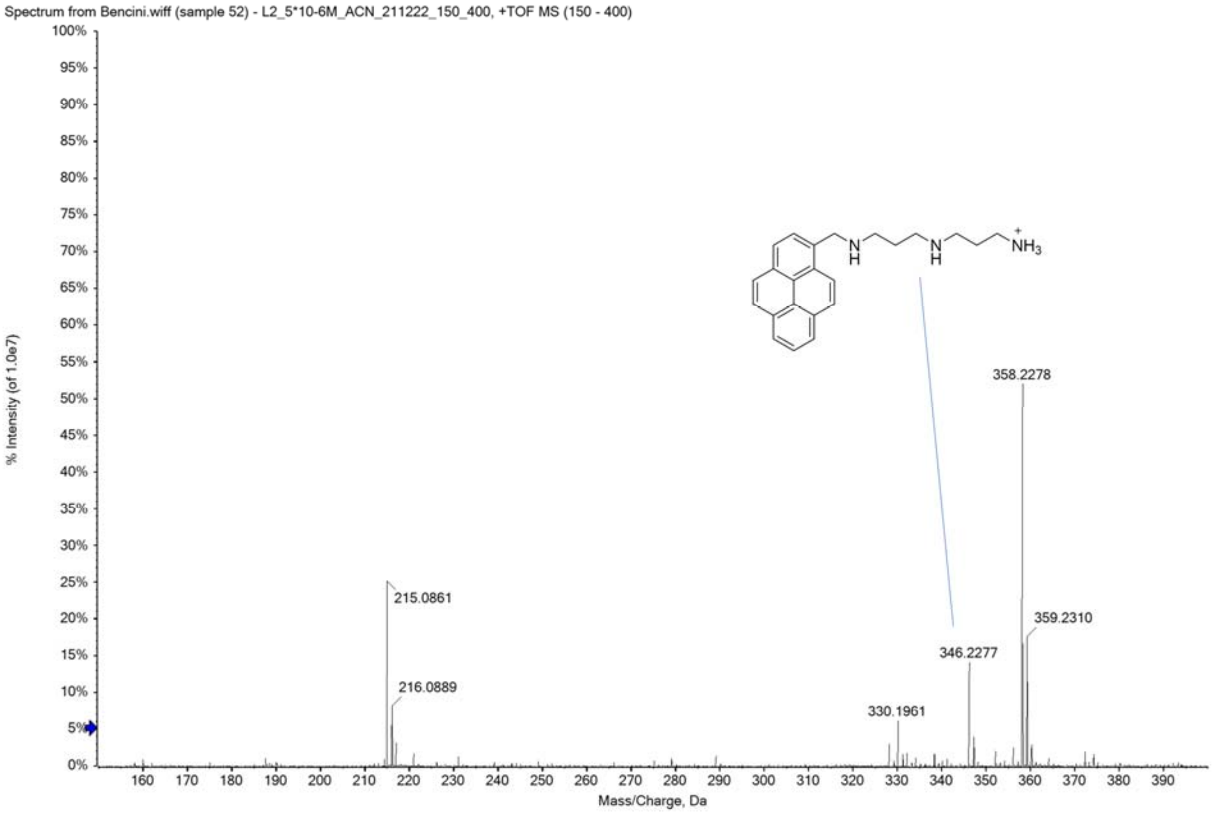

Figure S11. High resolution mass spectrum of compound **L2** in CH_3CN; 346.2277 (z = 1, [**L1** + H]$^+$).

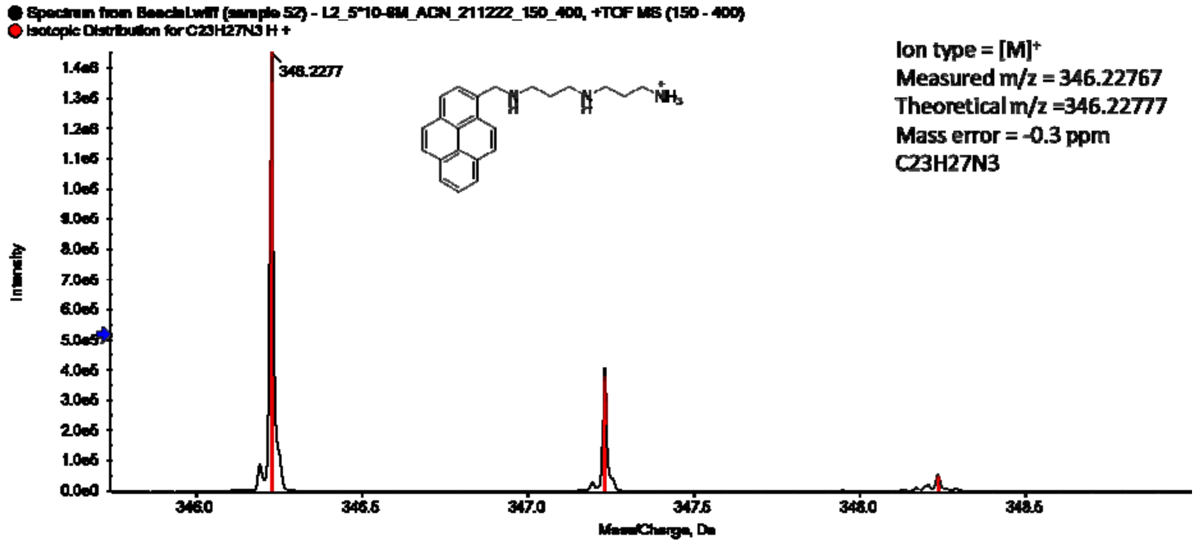

Figure S12. Isotopic pattern of the [**L2**+H]$^+$ (z = 1) ion, with measured (black) and theoretical (red) m/z value of the most abundant isotopic peak, 345.7-349.1 Da region.

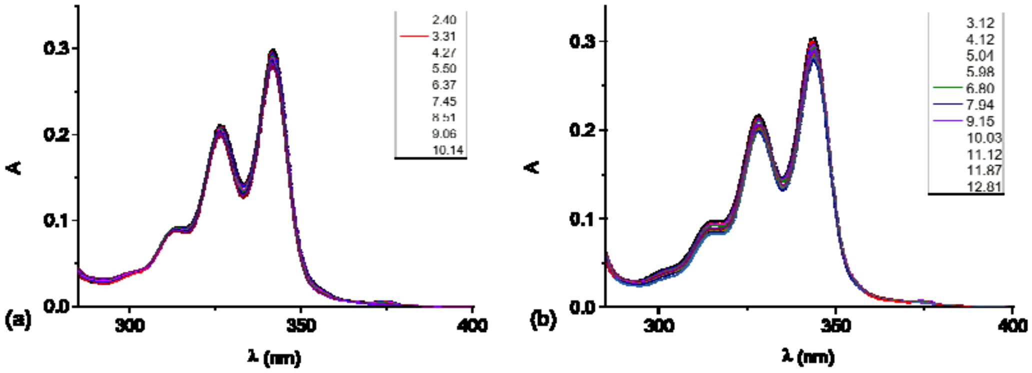

Figure S13. Absorption spectra of (a) **L1** and (b) **L2** at different pH values in H_2O/EtOH (50:50 v/v). ([**L1**] = [**L2**] = 10^{-5} M)

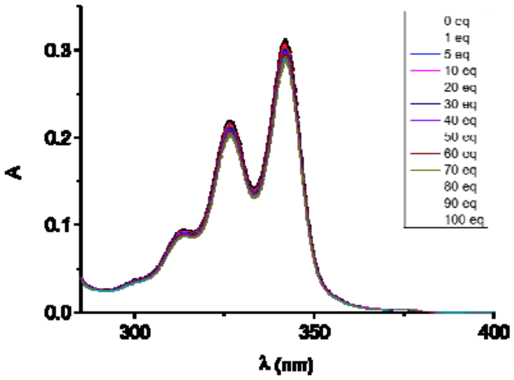

Figure S14. Absorption spectra of **L1** in H_2O/EtOH (50:50 v/v) at pH 7 (0.005 M TRIS buffer) in the presence of increasing amount of PFOA. ([**L1**] = 10^{-5} M)

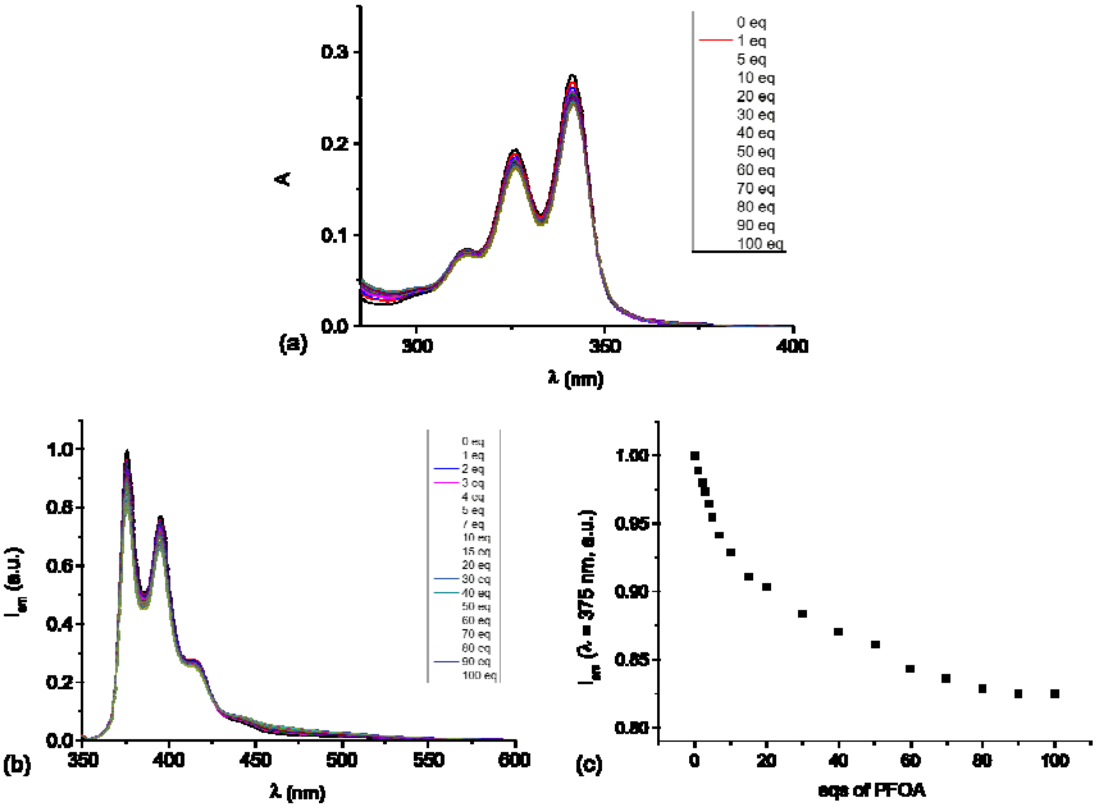

Figure S15. (a) Absorption and (b) emission spectra of **L2** in H_2O/EtOH (50:50 v/v) at pH 7 (0.005 M TRIS buffer) in the presence of increasing amount of PFOA. (c) Plot of the fluorescence emission of **L2** at 375 nm. ([**L2**] = 10^{-5} M, λ_{exc} = 340 nm)

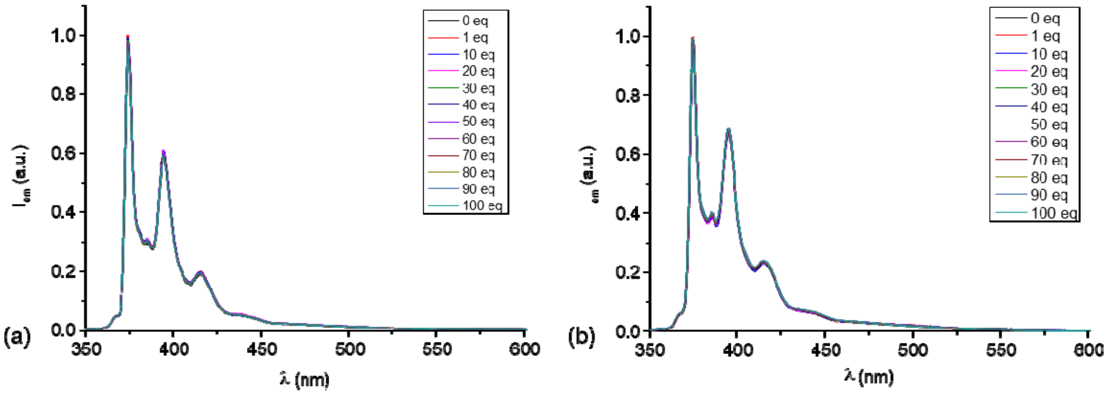

Figure S16. Emission spectra of (a) **L1** and (b) **L2** in H_2O/EtOH (50:50 v/v) at pH 7 (0.005 M TRIS buffer) in the presence of increasing amount of PFOS ([**L1**] = [**L2**] = 10^{-5} M, λ_{exc} = 340 nm).

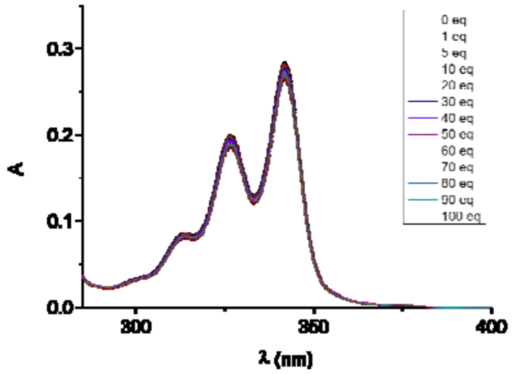

Figure S17. Absorption spectra of **L1** in H_2O/EtOH (50:50 v/v) at pH 4 in the presence of increasing amount of PFOA. ([**L1**] = 10^{-5} M)

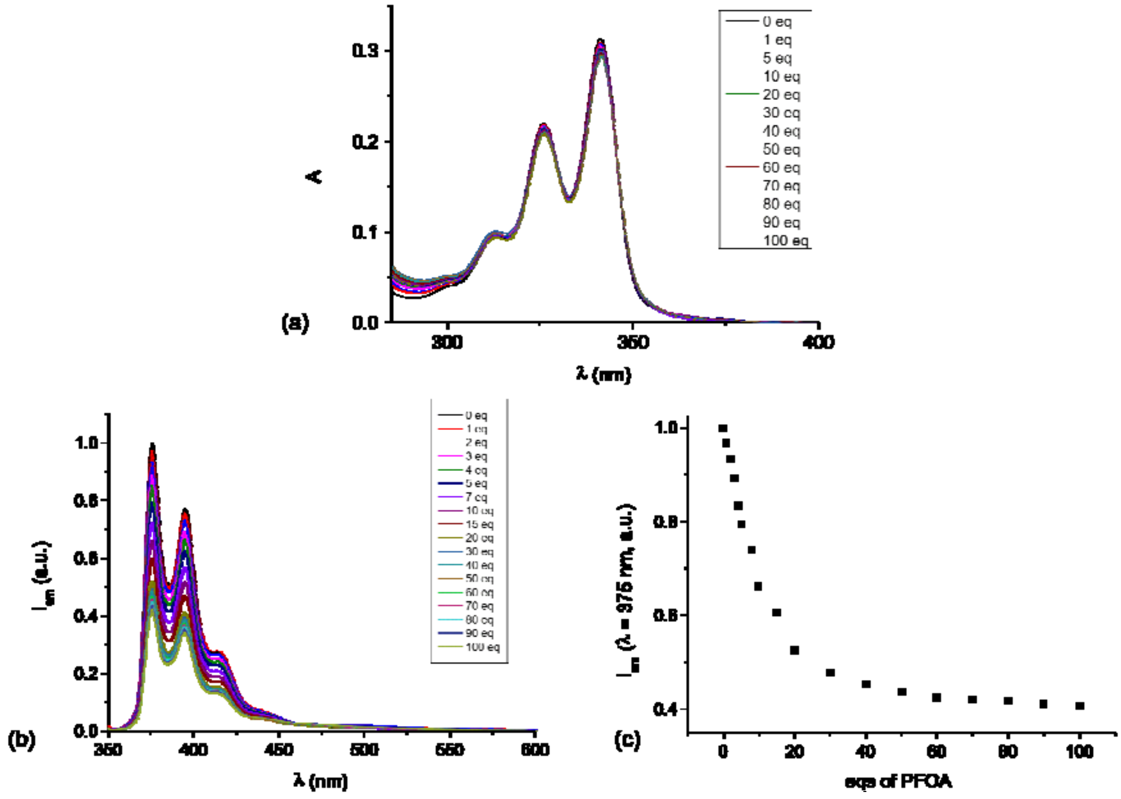

Figure S18. (a) Absorption and (b) emission spectra of **L2** in H_2O/EtOH (50:50 v/v) at pH 4 in the presence of increasing amount of PFOA. (c) Plot of the fluorescence emission of **L2** at 375 nm. ([**L2**] = 10^{-5} M, λ_{exc} = 340 nm)

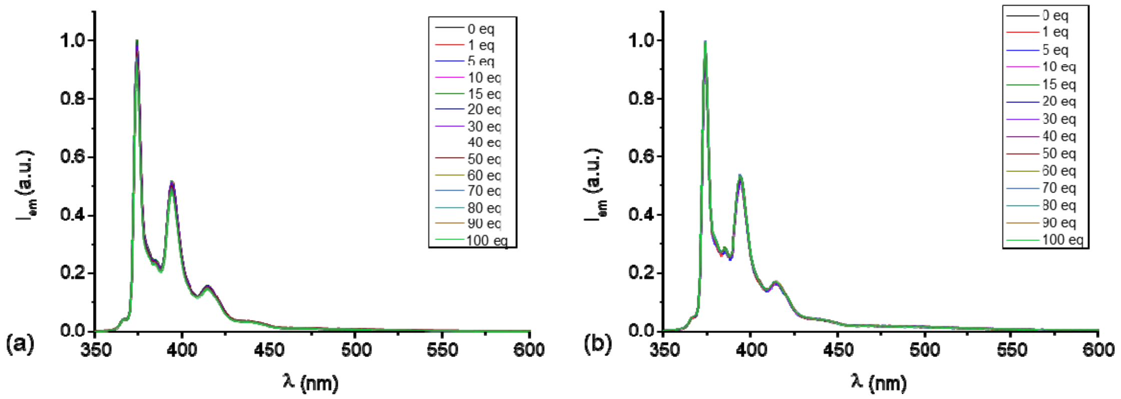

Figure S19. Emission spectra of (a) **L1** and (b) **L2** in H_2O/EtOH (50:50 v/v) at pH 4 in the presence of increasing amount of PFOS ([**L1**] = [**L2**] = 10^{-5} M, λ_{exc} = 340 nm).

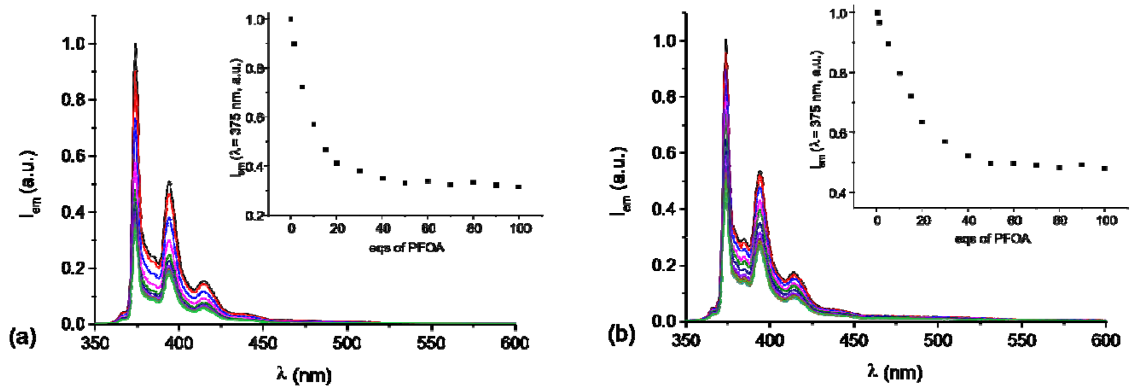

Figure S20. Emission spectra of (a) **L1** and (b) **L2** in H_2O/EtOH (50:50 v/v) at pH 4 at 308 K in the presence of increasing amount of PFOA ([**L1**] = [**L2**] = 10^{-5} M, λ_{exc} = 340 nm).

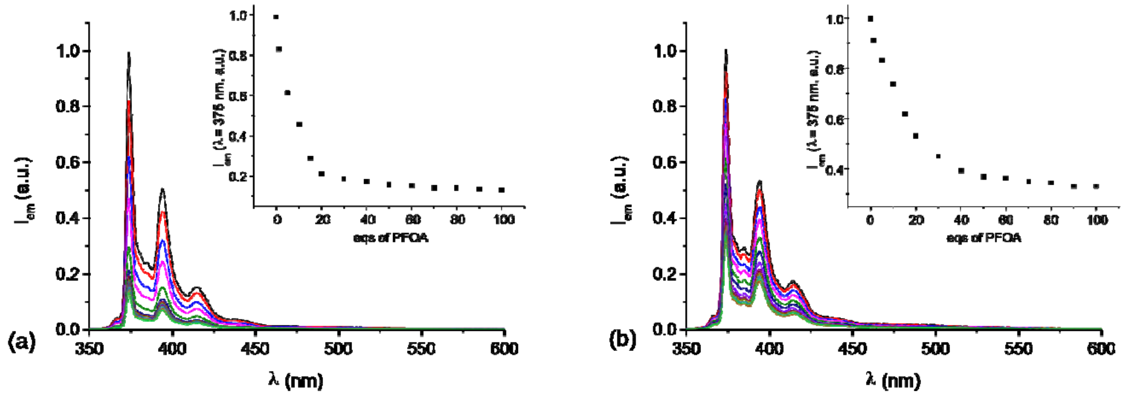

Figure S21. Emission spectra of (a) **L1** and (b) **L2** in H_2O/EtOH (50:50 v/v) at pH 4 at 288 K in the presence of increasing amount of PFOA ([**L1**] = [**L2**] = 10^{-5} M, λ_{exc} = 340 nm).

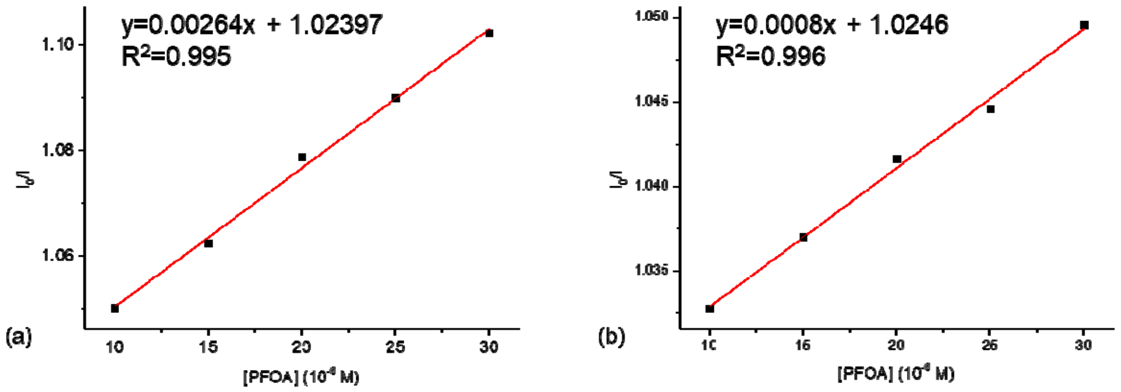

Figure S22. Plot of fluorescence emission I_0/I of (a) **L1** ([**L1**] =10^{-5} M, λ_{exc}=340 nm, s=0.001015) and (b) **L2** ([**L2**] = 10^{-5} M, λ_{exc} = 340 nm, s = 0.00182) at pH 7 (0.005 M TRIS buffer) in H_2O/EtOH 50:50 (v/v) in the presence of increasing amount of PFOA at 298 K. The detection limit was calculated to be 1.15 μM for **L1** and 6.8 μM for **L2**.

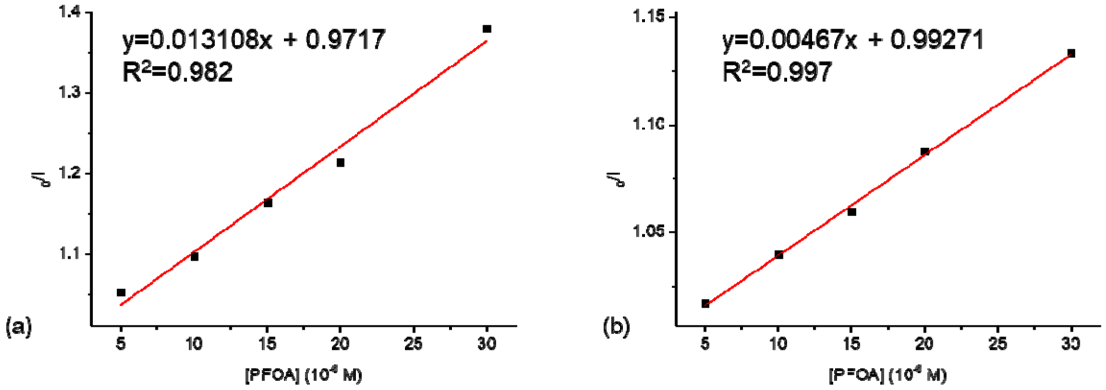

Figure S23. Plot of fluorescence emission I_0/I of (a) **L1** ([**L1**] =10^{-5} M, λ_{exc}=340 nm, s=0.0009814) and (b) **L2** ([**L2**] = 10^{-5} M, λ_{exc} = 340 nm, s = 0.0010214) at pH 4 in H_2O/EtOH 50:50 (v/v) in the presence of increasing amount of PFOA at 298 K. The detection limit was calculated to be 0.22 μM for **L1** and 0.66 μM for **L2**.

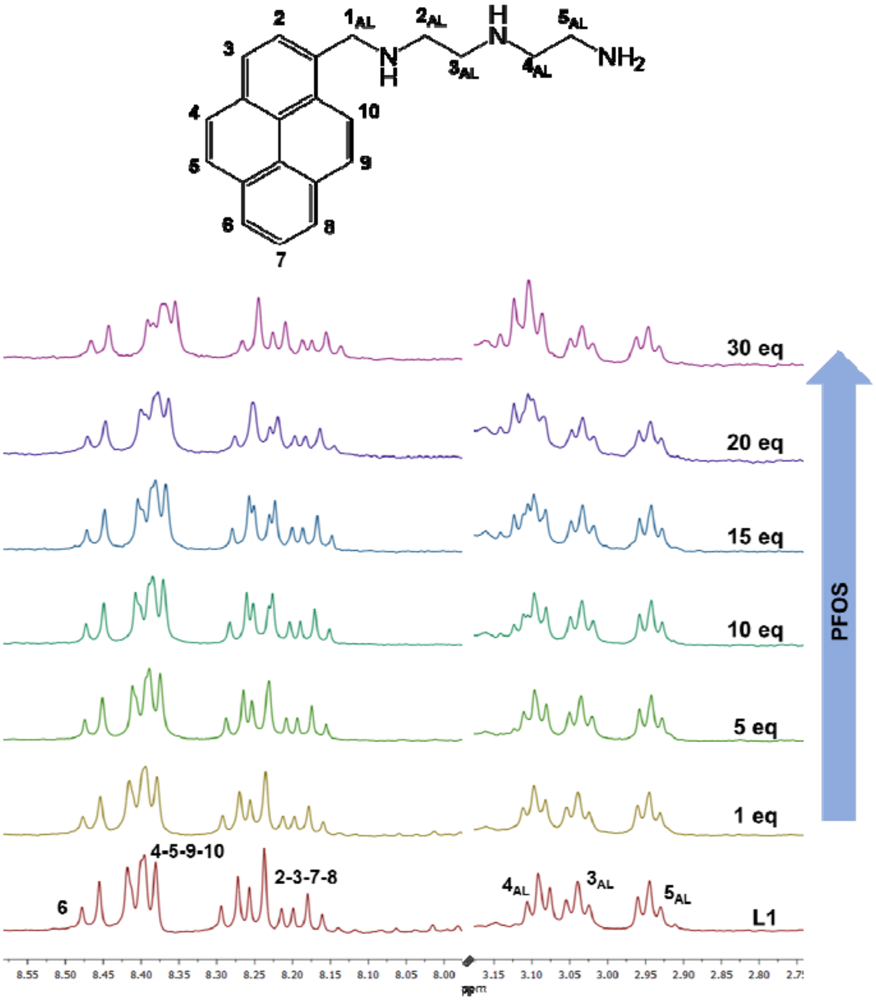

Figure S24. ^{1}H NMR spectra of **L1** at pH 4 D_2O/CD_3OD 40:60 (v/v) in the presence of increasing amounts of PFOS at 298 K. The signals of the 1_{AL} and 2_{AL} methylene groups cannot be observed due to overlapping with water and the methyl group of methanol, respectively.

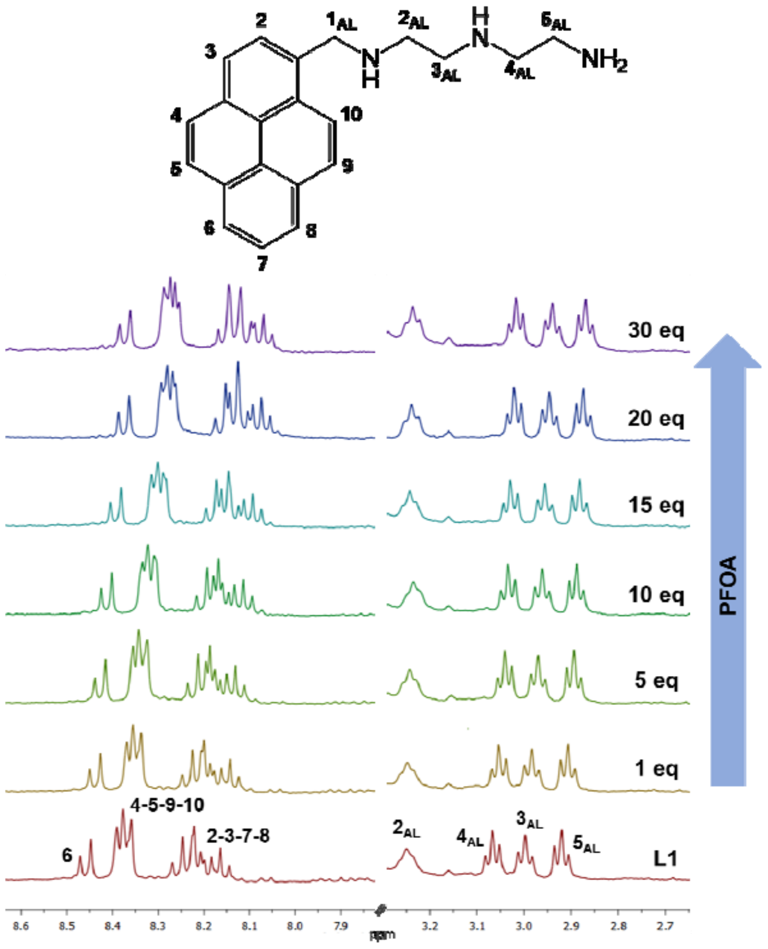

Figure S25. ^{1}H NMR spectra of **L1** at pH 7 D_2O/CD_3OD 40:60 (v/v) in the presence of increasing amounts of PFOA at 298 K. The signals of the 1_{AL} methylene group cannot be observed due to overlapping with water.

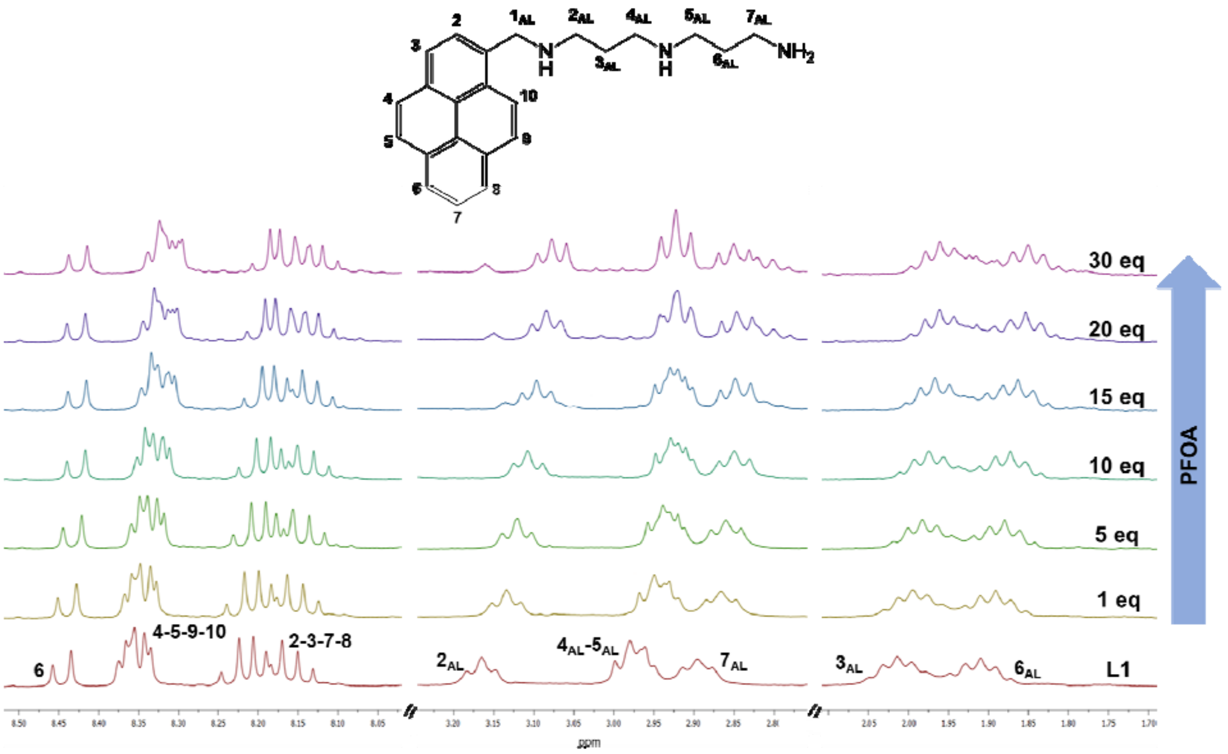

Figure S26. ^{1}H NMR spectra of **L2** at pH 7 D_2O/CD_3OD 40:60 (v/v) in the presence of increasing amounts of PFOA at 298 K. The signals of the 1_{AL} methylene group cannot be observed due to overlapping with water.

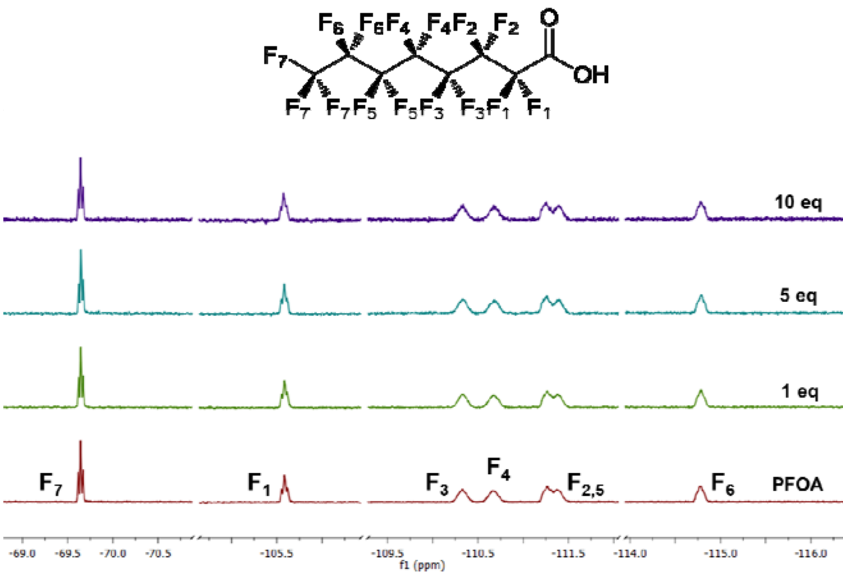

Figure S27. ^{19}F NMR spectra of PFOA at pH 7 D_2O/CD_3OD 40:60 (v/v) in the presence of increasing amounts of **L1** at 298 K.

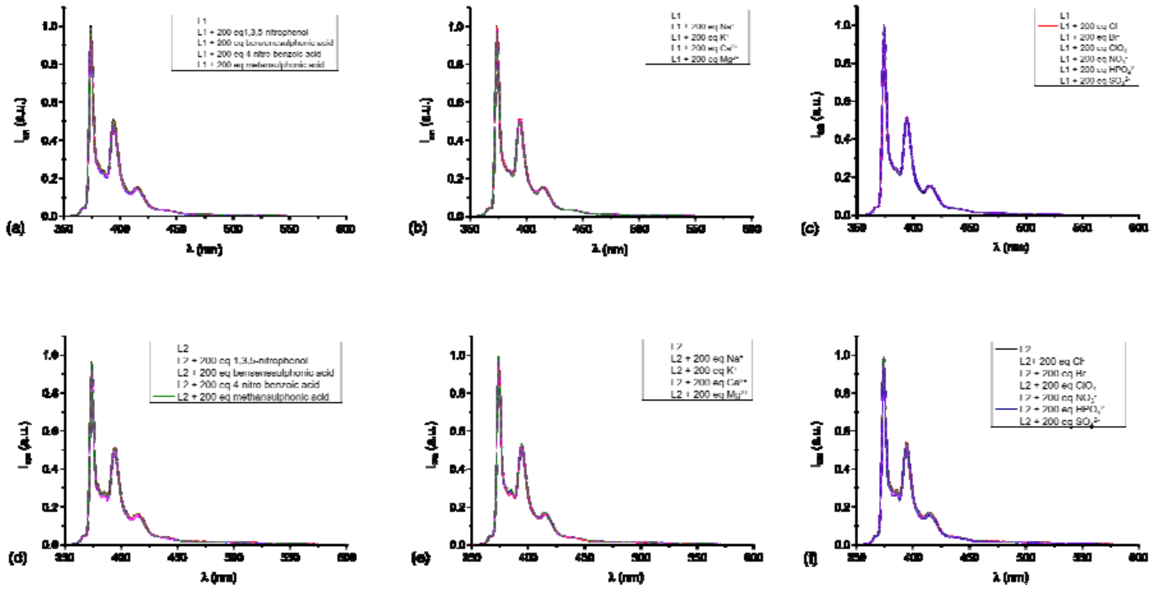

Figure S28. Emission spectra of **L1** (a-c) and **L2** (d-f) at pH 7 (0.005 M TRIS buffer, 298 K) in H_2O/EtOH 50:50 (Vol/Vol) in the absence and in the presence of 200 equivs. of different interfering agents. ([**L1**] = 10^{-5} M, [**L2**] = 10^{-5} M, λ_{exc} = 340 nm); a) **L1** + 1,3,5-nitrophenol, benzenesulphonic acid, 4-nitro-benzoic acid or methansulphonic acid; b) **L1** + Na^+, K^+, Mg^{2+} or Ca^{2+}; c) **L1** + Cl^-, Br^-, ClO_4^-, NO_3^-, HPO_4^{2-} or SO_4^{2-}; d) **L2** + 1,3,5-nitrophenol, benzenesulphonic acid, 4-nitro-benzoic acid or methansulphonic acid; e) **L2** + Na^+, K^+, Mg^{2+} or Ca^{2+}; f) **L2** + Cl^-, Br^-, ClO_4^-, NO_3^-, HPO_4^{2-} or SO_4^{2-} (pH 7, 0.005 M TRIS buffer, 298 K, H_2O/EtOH 50:50 (Vol/Vol)). The pH of the buffer was generally adjusted at 7 by addition of NaOH to a solution of TRIS.HCl. In the test with Na^+ as interfering agent the TRIS buffer at pH 7 was prepared starting form a TRIS.HCl solution and adjusting the pH at 7 with NMe_4OH, to avoid the presence of Na^+ in the tested solution; in the test with Cl- as interfering agent the TRIS buffer at pH 7 was prepared by using TRIS free base and adjusting the pH at 7 with CF_3SO_3H, to avoid the presence of Cl in the tested solution.

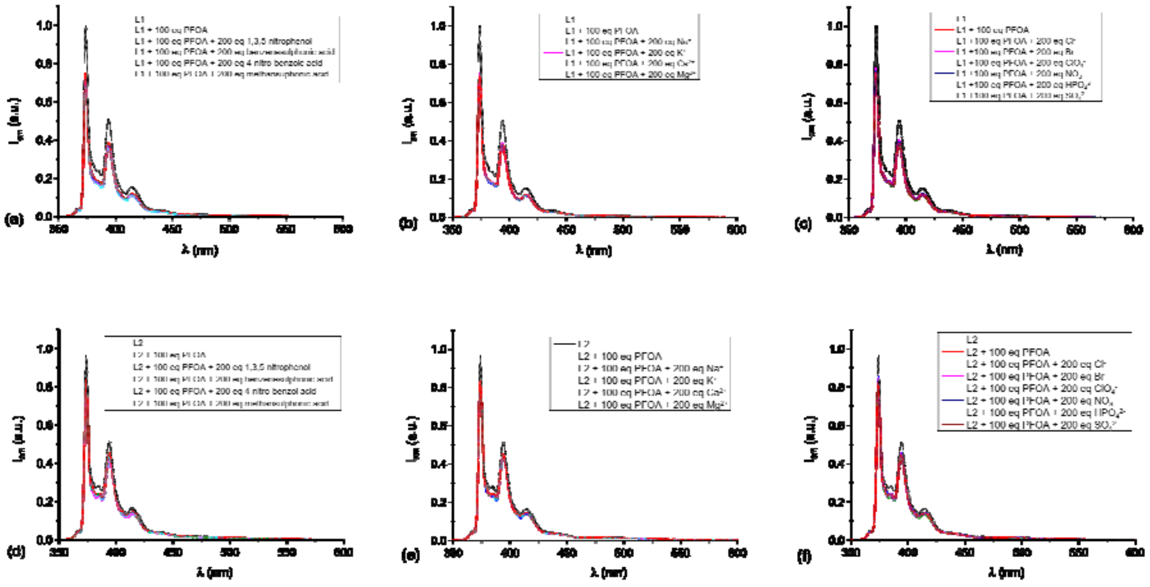

Figure S29. Emission spectra of **L1** (a-c) and **L2** (d-f), **L1** and **L2** with 100 equivs. of PFOA and **L1** and **L2** with 100 equivs. of PFOA in the presence of 200 equivs. of different interfering agents at pH 7 (0.005 M TRIS buffer, 298 K) in H_2O/EtOH 50:50 (Vol/Vol) ([**L1**] = 10^{-5} M, [**L2**] = 10^{-5} M, λ_{exc} = 340 nm); a) **L1** + PFOA + 1,3,5-nitrophenol, benzenesulphonic acid, 4-nitro-benzoic acid or methansulphonic acid; b) **L1** + PFOA + Na^+, K^+, Mg^{2+} or Ca^{2+}; c) **L1** + PFOA + Cl^-, Br^-, ClO_4^-, NO_3^-, HPO_4^{2-} or SO_4^{2-}; d) **L2** + PFOA + 1,3,5-nitrophenol, benzenesulphonic acid, 4-nitro-benzoic acid or methansulphonic acid; e) **L2** + PFOA + Na^+, K^+, Mg^{2+} or Ca^{2+}; f) **L2** + PFOA + Cl^-, Br^-, ClO_4^-, NO_3^-, HPO_4^{2-} or SO_4^{2-} (pH 7, 0.005 M TRIS buffer, 298 K, H_2O/EtOH 50:50 (Vol/Vol)). The pH of the buffer was generally adjusted at 7 by addition of NaOH to a solution of TRIS.HCl. In the test with Na^+ as interfering agent the TRIS buffer at pH 7 was prepared starting form a TRIS.HCl solution and adjusting the pH at 7 with NMe_4OH, to avoid the presence of Na^+ in the tested solution; in the test with Cl- as interfering agent the TRIS buffer at pH 7 was prepared by using TRIS free base and adjusting the pH at 7 with CF_3SO_3H, to avoid the presence of Cl in the tested solution.

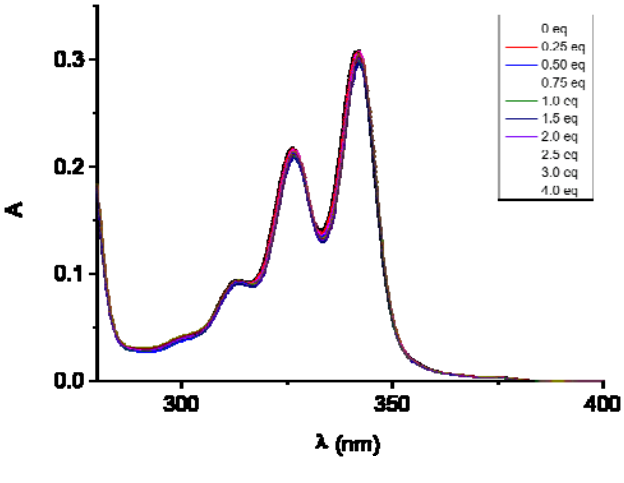

Figure S30. Absorption spectra of **L1** in H_2O/EtOH (50:50 v/v) at pH 8 (0.005 M TRIS buffer) in the presence of increasing amount of Zn(II). ([**L1**] = 10^{-5} M)

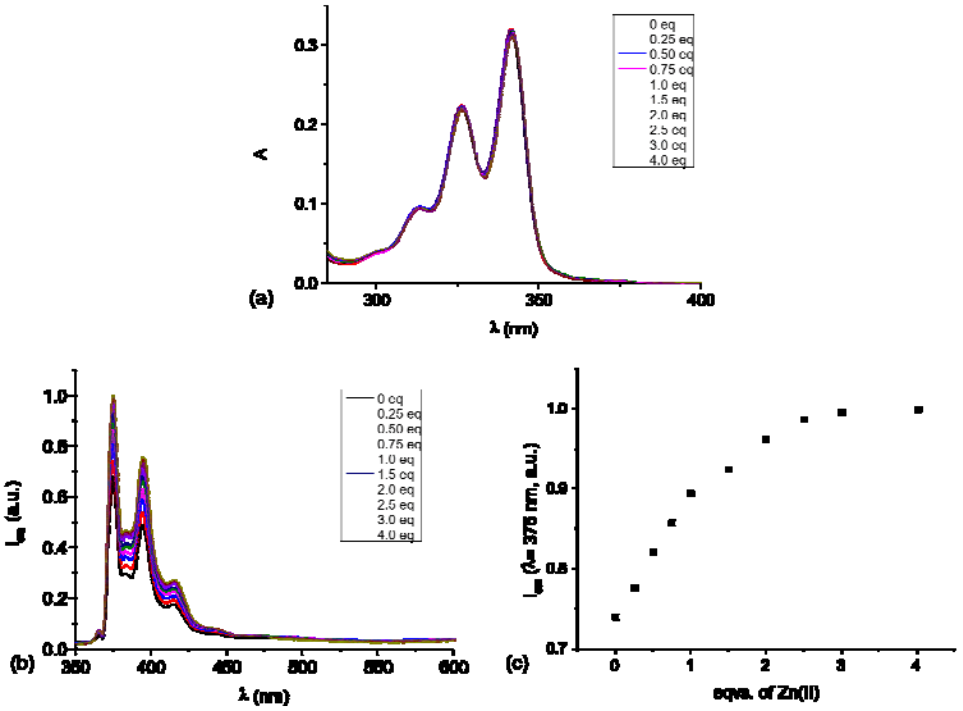

Figure S31. (a) Absorption and (b) emission spectra of **L2** in H_2O/EtOH (50:50 v/v) at pH 8 (0.005 M TRIS buffer) in the presence of increasing amount of Zn(II). (c) Plot of the fluorescence emission of **L2** at 375 nm. ([**L2**] = 10^{-5} M, λ_{exc} = 340 nm)

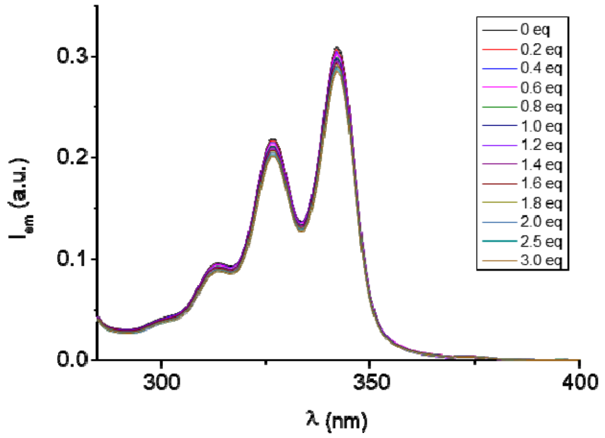

Figure S32. Absorption spectra of $[ZnL1]^{2+}$ in H_2O/EtOH (50:50 v/v) at pH 8 (0.005 M TRIS buffer) in the presence of increasing amount of PFOA. ([$[ZnL1]^{2+}$] = 10^{-5} M)

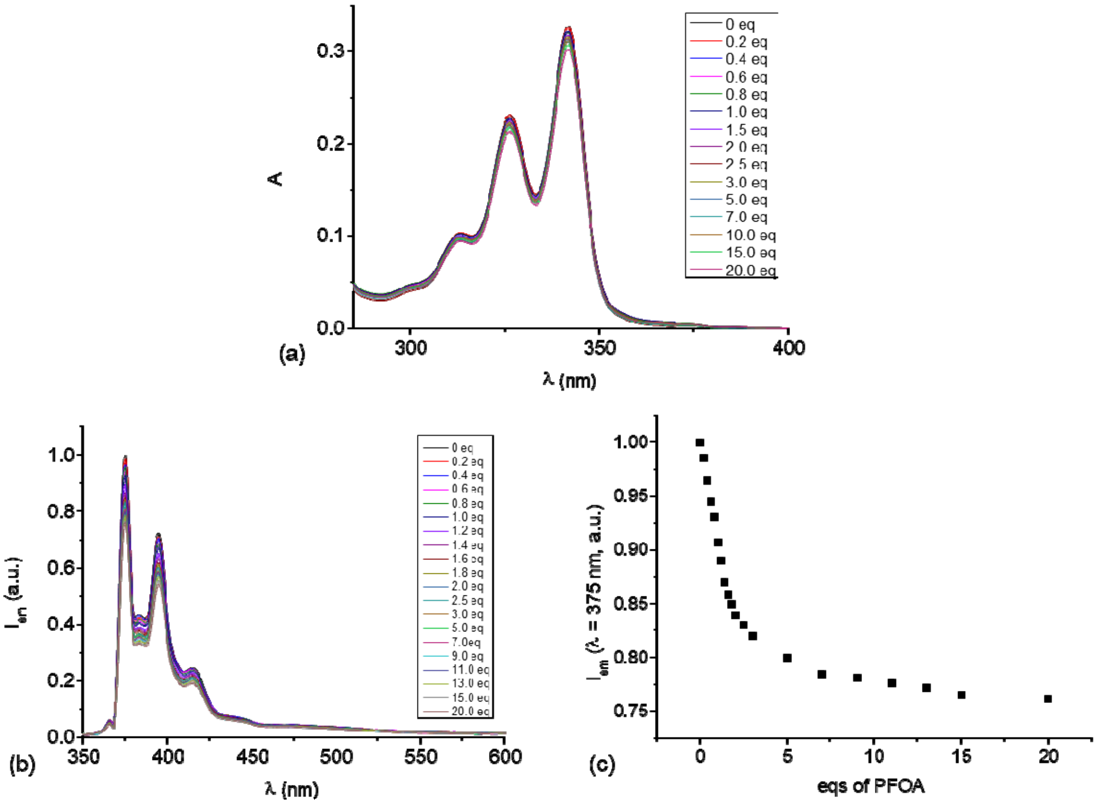

Figure S33. (a) Absorption and (b) emission spectra of [Zn**L2**]$^{2+}$ in H_2O/EtOH (50:50 v/v) at pH 8 (0.005 M TRIS buffer) in the presence of increasing amount of PFOA. (c) Plot of the fluorescence emission of [Zn**L2**]$^{2+}$ at 375 nm. ([Zn**L2**]$^{2+}$ = 10^{-5} M, λ_{exc} = 340 nm)

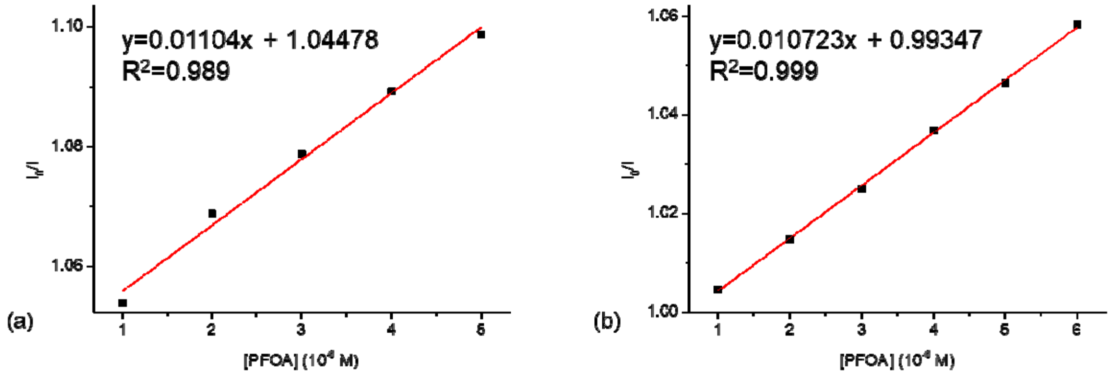

Figure S34. Plot of fluorescence emission I_0/I of (a) [Zn**L1**]$^{2+}$ ([[Zn**L1**]$^{2+}$] =10^{-5} M, λ_{exc}=340 nm, s=0.022915) and (b) [Zn**L2**]$^{2+}$ ([[Zn**L2**]$^{2+}$] =10^{-5} M, λ_{exc}=340 nm, s=0.039915) at pH 8 (0.005 M TRIS buffer) in H_2O/EtOH 50:50 (v/v) in the presence of increasing amount of PFOA at 298 K. The detection limit was calculated to be 0.62 μM for Zn**L1** and 11.2 μM for Zn**L2**.

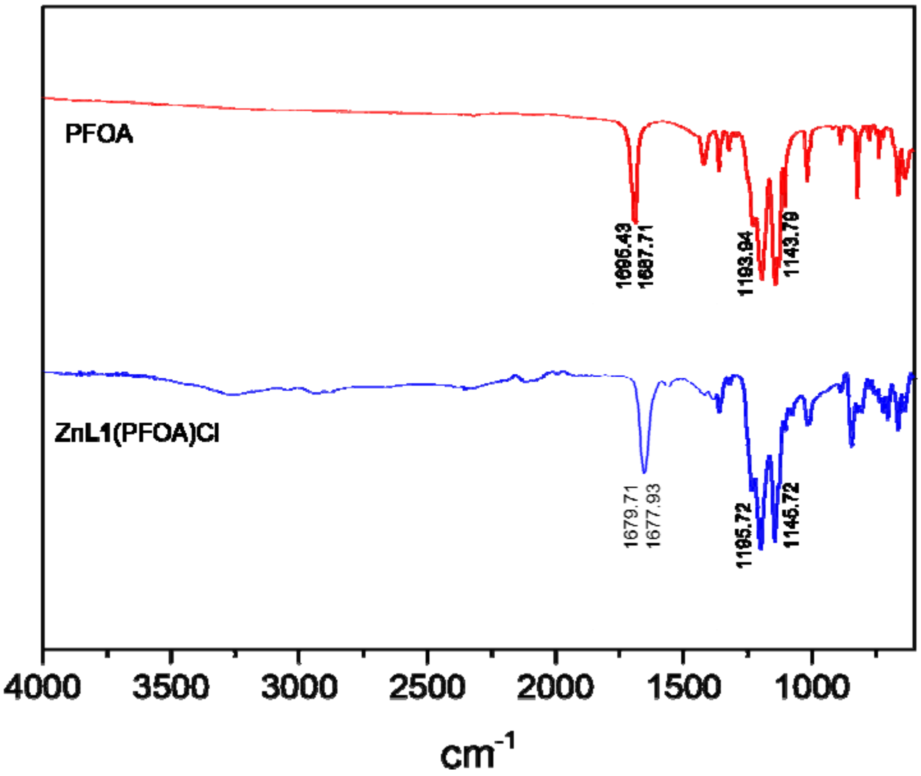

Figure S35. FT-IR solid state spectra of PFOA sodium salt and the Zn**L1**(PFOA)Cl complex.

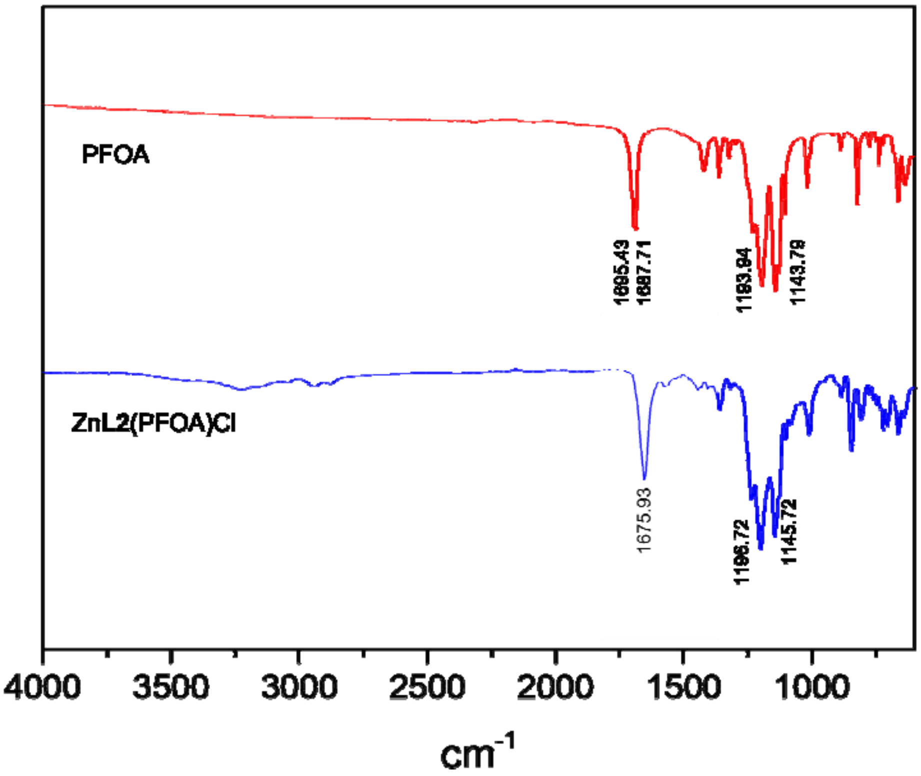

Figure S36. FT-IR solid state spectra of PFOA sodium salt and the Zn**L2**(PFOA)Cl complex.

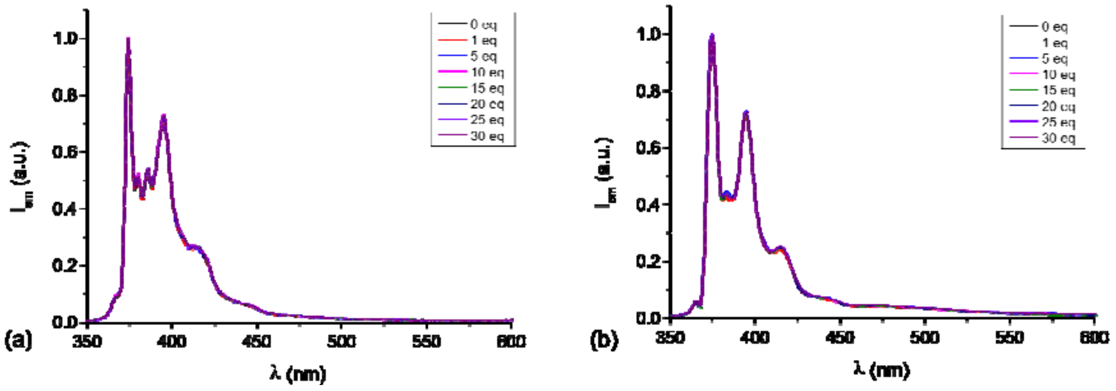

Figure S37. Emission spectra of (a) [Zn**L1**]$^{2+}$ and (b) [Zn**L2**]$^{2+}$ in H_2O/EtOH (50:50 v/v) at pH 8 in the presence of increasing amount of PFOS ([[Zn**L1**]$^{2+}$] = [[Zn**L2**]$^{2+}$] = 10^{-5} M, λ_{exc} = 340 nm).

Table S1. Bond distances and angles defining the Zn(II) coordination environment in the crystal structure of **6**

**Bond Distances**		**Bond Angles**	
Zn1- N1	2.012(2)	Cl1-Zn1-N1	107.02(5)

Zn1- N2	2.035(1)	Cl1-Zn1-N2	109.18(5)
Zn1- N3	2.042(2)	Cl1-Zn1-N3	106.82(5)
Zn1- Cl1	2.2420(5)	N1-Zn1-N2	98.61(7)
		N2-Zn1-N3	98.46(7)
		N3-Zn1-N1	134.19(7)

Table S2. Crystallographic data and refinement parameters for **6**

	**6**
Formula	$[Zn(C_{23}H_{25}N_3)Cl]ClO_4$
M	543.73
T (K)	100
λ (Å)	1.54184
Crystal system, space group	Triclinic, P-1
Unit cell dimensions (Å, °)	a =8.7070(2); α = 77.662(1)<br>b = 11.4358(2); β = 86.278(1)<br>c = 12.7837(3); γ = 68.582(1)
V (Å^3)	1157.50(4)
Z, ρ (mg/cm3)	2, 1.560
μ (mm^{-1})	3.903
F(000)	560
Crystal size	0.17x0.18x0.20
θ range (°)	3.539-72.429
Reflns collected / unique (R_{int})	33464 / 4558 (0.0448)
Data / parameters	4558 / 379
Final R indices [I > 2σ]	0.0281 / 0.0803
R indices (all data)	0.0305 / 0.0834
GoF	0.884
